# Efficient Capture of Short‐ and Long‐Chain PFAS from Water by a Metal–Organic Framework

**DOI:** 10.1002/smll.202510000

**Published:** 2025-11-06

**Authors:** Thais Grancha, Patricia García‐Atienza, Lidia García, Sergio Armenta, José Manuel Herrero‐Martínez, Donatella Armentano, Teresa F. Mastropietro, Jesús Ferrando Soria, Emilio Pardo

**Affiliations:** ^1^ Instituto de Ciencia Molecular (ICMol) Universidad de Valencia Paterna Valencia 46980 Spain; ^2^ Departamento de Química Analítica Universitat de València c/Dr. Moliner, 50 Burjassot Valencia 46100 Spain; ^3^ Dipartimento di Chimica e Tecnologie Chimiche (CTC) Università della Calabria Rende Calabria 87036 Italy

**Keywords:** metal–organic frameworks, PFAS, single crystal X‐ray diffraction, water remediation

## Abstract

Per‐ and polyfluoroalkyl substances (PFAS), known as “forever chemicals,” present major environmental and health risks due to their extreme stability and dual hydrophobic–hydrophilic character, which complicates remediation. Conventional adsorbents such as activated carbon and ion‐exchange resins show limited performance, particularly for short‐chain PFAS. Metal–organic frameworks (MOFs) have emerged as promising alternatives owing to their tunable porosity, large surface area, and adjustable functionality. Here, we assess the PFAS removal potential of a robust, water‐stable, biologically derived MOF, Cu^II^_2_(*S*,*S*)‐hismox·5H_2_O (denoted **1**), synthesized from *L*‐histidine. MOF **1** features medium‐sized trapezoidal nanoscale channels exhibiting both hydrophobic and hydrophilic character. It achieved high capture efficiencies (80–100%) for long‐chain PFAS (C₇–C₁_2_), including PFDA, PFUnDA, PFDoDA, PFOS, and 8:2 FTSA, and remarkable removal rates of 70% (PFBA) and 86% (PFBS) for short‐chain analogues –surpassing conventional adsorbents and other reported MOFs. Excellent reusability and rapid adsorption kinetics were observed under continuous‐flow solid‐phase extraction with contact times under 30 seconds. The high crystallinity of MOF **1** also enabled single‐crystal X‐ray diffraction studies of encapsulated PFBA and PFOS (**PFBA@1** and **PFOS@1**). These findings highlight MOF **1** as a high‐performance, bio‐derived platform for efficient PFAS remediation and advance the development of MOF‐based water treatment technologies.

## Graphical abstract

A biologically derived metal‐organic framework (MOF) shows rapid and efficient removal and extraordinary reusability of both long‐ and short‐chain PFAS, outperforming conventional adsorbents. With dual hydrophobic‐hydrophilic channels and fast adsorption under continuous‐flow conditions, this MOF enables structural insights via X‐ray diffraction and offers a promising solution for advanced water remediation.

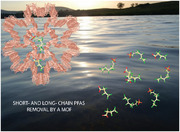

## 1 Introduction

Per‐ and polyfluoroalkyl substances (PFAS)^[^ ^1^, ^2^ ^]^ are synthetic chemicals defined by their exceptionally strong carbon‐fluorine bonds, which grant them remarkable resistance to degradation.^[^ ^3^, ^4^, ^5^ ^]^ These compounds are extensively used (and produced) in various industries and consumer products, including non‐stick cookware, firefighting foams, and water‐resistant fabrics.^[^ ^6^ ^]^ Indeed, due to their environmental persistence, PFAS are often referred to as “forever chemicals”. As a consequence, they can accumulate in water, soil, and living organisms, resulting in bioaccumulation and biomagnification throughout the food chain.^[^ ^7^, ^8^, ^9^ ^]^ Human exposure to PFAS has been linked to severe health concerns, such as cancer, liver damage, immune system disruption, and developmental disorders, posing a serious risk to public health and environmental systems.^[^ ^10^ ^]^ Thus, it is clear that the discharge/production of these compounds must be minimized as much as possible. In this regard, various regulatory bodies have already begun to introduce legislation addressing this issue. For example, a new European Directive^[^ ^11^ ^]^ limits the total amount of PFAS that can be present in discharged aqueous solutions to 0.5 ppb, which is far beyond the reach of current technologies (*vide infra*).

The effective removal of PFAS from the environment presents a significant challenge due to their exceptional chemical stability and also their dual chemical nature, as they possess a hydrophobic tail (carbon‐fluoride chain) but also a hydrophilic head (sulfonic or carboxylic acid, alcohol, etc.).^[^ ^12^ ^]^ Remediation strategies for PFAS mainly focus on two methods: capture and degradation. However, degradation is particularly challenging because PFAS are resistant to chemical breakdown, and partial degradation can generate harmful by‐products.^[^ ^3^ ^]^ As a result, capture has emerged as a more feasible alternative. Commonly used adsorbent materials for PFAS removal from water include granular (GAC) and powdered (PAC) activated carbons,^[^ ^13^, ^14^, ^15^, ^16^ ^]^ covalent organic frameworks (COFs),^[^ ^17^ ^]^ single‐walled carbon nanotubes (SWCNT),^[^ ^18^ ^]^ ion‐exchange resins,^[^ ^19^, ^20^ ^]^ zeolites^[^ ^21^ ^]^ and innovative options like biochar^[^ ^22^ ^]^ or chitosan‐molecular imprinted polymers.^[^ ^23^ ^]^ These materials work by trapping PFAS on their surfaces and/or channels, effectively preventing their reintroduction into the environment. Capture does not only facilitate safer disposal but also enables the potential reuse of adsorbents, making it a more practical and manageable solution compared to degradation. However, the PFAS capture performance of these types of materials has been, at best, only moderate. In particular, the results for short‐chain PFAS capture^[^ ^24^ ^]^ –typically those with 4 ≤ C < 6 for perfluoroalkyl sulfonates and 5 ≤ C < 8 for perfluoroalkyl carboxylates– remain far from satisfactory.^[^ ^13^, ^24^ ^]^ This is likely due to their distinctive dual chemical nature,^[^ ^2^ ^]^ characterized by the simultaneous presence of hydrophobic and hydrophilic domains within the same molecule. This amphiphilic structure results in high water solubility and weak interactions with conventional adsorbent materials. In summary, it is clear that further efforts are needed to understand the mechanisms governing the capture of these species, which exhibit both hydrophobic and hydrophilic properties within the same molecule.^[^ ^1^, ^2^ ^]^ Ultimately, this knowledge will enable the design of more effective adsorbents.

Metal–organic frameworks^[^ ^25^, ^26^, ^27^, ^28^ ^]^ (MOFs) are a particular type of crystalline porous materials that have shown great potential in water remediation^[^ ^29^, ^30^ ^]^ due to their high surface area, tunable porosity, and ability to be functionalized for specific targets.^[^ ^31^ ^]^ These properties situate them, in principle, among the most promising candidates for capturing PFAS,^[^ ^32^, ^33^, ^34^ ^]^ as MOFs can be designed to selectively adsorb pollutants based on size, charge, and hydrophobicity. It is known that the amphiphilic nature of PFAS,^[^ ^1^, ^2^ ^]^ combined with their chemical resistance, makes conventional adsorbents less efficient, but MOFs can be engineered to address these challenges.^[^ ^35^ ^]^ Their customizable pore structures and surface functionalities could allow MOFs to target and capture a wide range of PFAS molecules, making them an attractive alternative for improving the efficiency of PFAS removal from contaminated water. Indeed, a certain number of recent works^[^ ^36^, ^37^, ^38^, ^39^, ^40^, ^41^, ^42^, ^43^, ^44^, ^45^, ^46^, ^47^, ^48^, ^49^, ^50^, ^51^, ^52^ ^]^ have unveiled –with varying degrees of success– their potential in the removal of long‐chain PFAS.^[^ ^36^, ^37^, ^38^, ^39^, ^45^, ^46^, ^47^, ^48^, ^49^, ^50^, ^51^, ^52^ ^]^ In contrast, very few studies address the issue of short chain PFAS.^[^ ^40^, ^41^, ^42^, ^43^, ^44^ ^]^ Certainly, their removal remains challenging due to their high polarity and hydrophilic nature, and further efforts are needed to design MOFs with suitable pore sizes and chemical environments.

## 2 Results and Discussion

**Figure 1:**
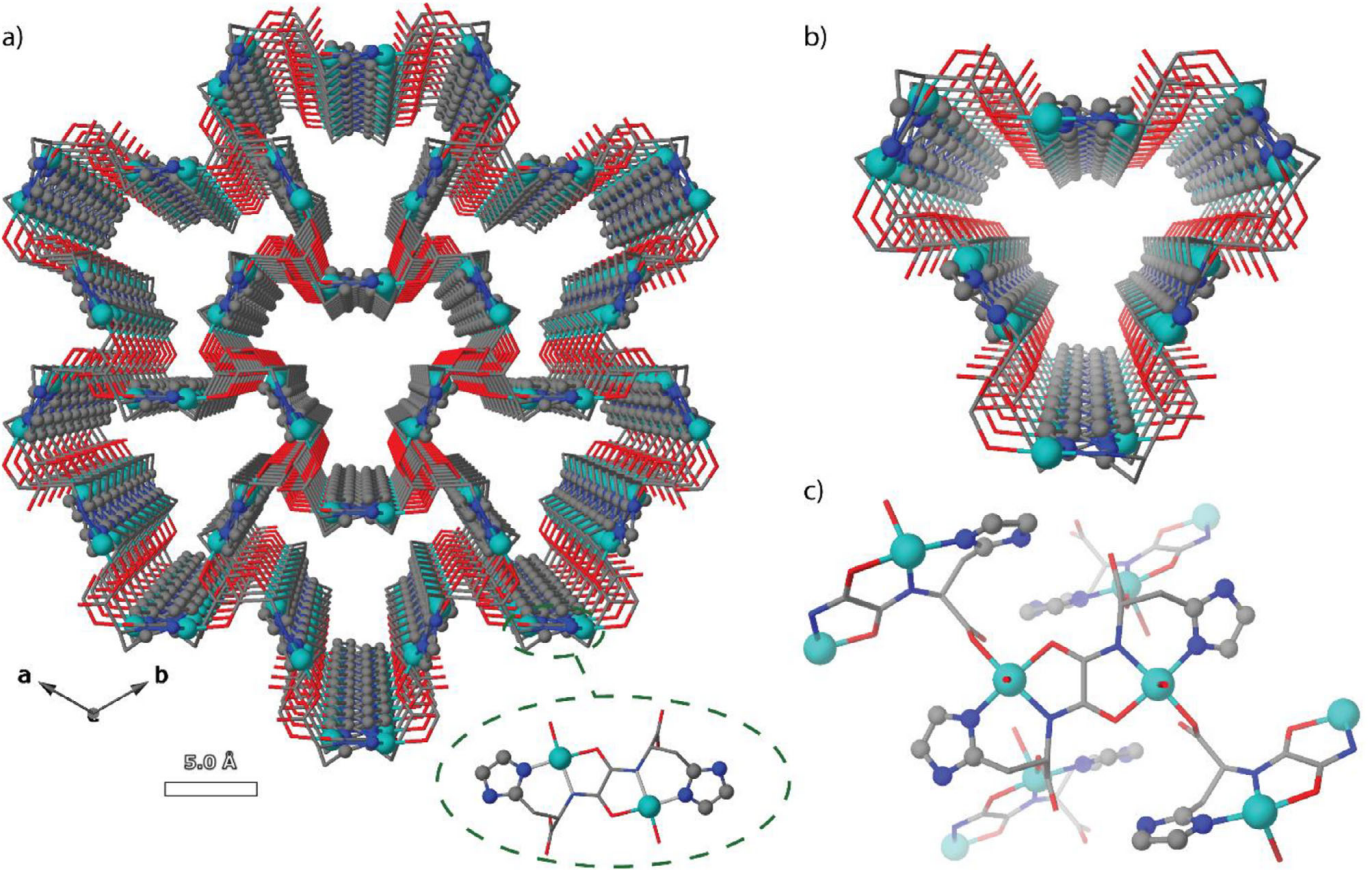
a) Perspective view of the 3D open‐framework of 1 along the *c* axis. b) Perspective view of one single channel of 1 along the *c* axis. c) Perspective view of a fragment of 1 in the *bc* plane, showing the dinuclear units coordination modes. The ligands are depicted as colored sticks, with the exception of imidazole rings which are represented as spheres, and the copper(II) cations as spheres, Color codes: Cu: cyan; O: red; C: gray; N: blue.

In this work, we investigate the potential of a previously reported biological MOF (bioMOF), formed by ligands derived from biomolecules –in this case, the amino acid *L*‐histidine– with the formula Cu^II^_2_(*S,S*)‐hismox ^.^ 5H_2_O^[^ ^53^ ^]^ (**1**) (**Figure** **1**), where hismox represents the oxamidato‐based^[^ ^54^, ^55^, ^56^, ^57^, ^58^, ^59^, ^60^ ^]^ ligand bis[(*S*)‐histidine]oxalyl diamide, for PFAS capture. This MOF was selected due to its unique characteristics, which could make it highly suitable for capturing PFAS. It features medium‐sized pores with a distinctive shape and contains niches where small species can fit and interact with copper(II) cations and/or imidazole rings. These channels exhibit a dual nature: a hydrophobic part, formed by the ligands constituting the framework, and a hydrophilic part, comprising small “pockets” that house the copper atoms and imidazole residues (Figure 1). Furthermore, MOF **1** had demonstrated, previously, reversible and continuous breathing, as evidenced by single‐crystal X‐ray diffraction, a property that can enhance host‐guest interactions with such small species as PFAS. These features were highlighted in a previous study, which showed that MOF **1** efficiently captured various gases (Ar, N_2_, CO_2_, and C_3_H_6_).^[^ ^53^ ^]^ The gases were found to occupy the “pockets” and were strongly stabilized, enabling the resolution of the crystal structures of the corresponding host‐guest aggregates. Based on these properties, we aim to evaluate the efficiency of this material in removing a series of both short‐ and long‐chain carboxylic and sulfonic acid PFAS from water (Scheme S1, Supporting Information).

### 2.1 Synthesis and Characterization

Prior to being used in capture experiments, MOF **1** was prepared and characterized, in a multigram scale, by following a synthetic method, previously reported.^[^ ^53^ ^]^ A gram‐scale synthesis of compound **1** was achieved by dissolving 2.5 g of the previously synthesized metalloligand (Bu_4_N)_2_{Cu_2_(S,S)‐hismox_2_}·4H_2_O in water, followed by gradual acidification with HCl (pH  =  2) under stirring to reach pH ≈ 4.0. The resulting blue polycrystalline solid was isolated by filtration, washed, and vacuum‐dried. Moreover, blue prisms of compound **1**, suitable for X‐ray diffraction experiments aimed at tentatively unveiling the host–guest structures of various PFAS‐loaded adsorbates, were obtained by slow diffusion of an acidic aqueous solution (pH  =  2) over a 2 mL aqueous layer of the precursor (Bu_4_N)_2_{Cu_2_(S,S)‐hismox_2_}·4H_2_O (0.1 mmol) in an assay tube. MOF **1** crystallizes in the chiral *P*3_1_21 space group of the hexagonal system (Table S1, Supporting Information). **1** exhibits a qtz‐e‐type topology. The framework comprises six connecting nodes of trans‐oxamidato‐bridged dicopper(II) units, {Cu^II^_2_[(*S*,*S*)‐hismox]} (inset of Figure 1a), bridged by the carboxylate of the histidine moieties acting as monodentate linkers, creating an infinite chiral porous 3D framework with unidimensional trapezoidal nanosized channels growing along the *c* axis (Figure 1), which seem suitable to host PFAS molecules, whose kinetic diameters are in the range 0.29–0.38 Å. Additionally, MOF **1** possesses accessible and unsaturated copper(II) centers, which can be coordinated by the carboxylates or sulfonates of the corresponding PFAS, as well as accessible imidazole groups, which could establish weak intermolecular interactions, such as hydrogen bonds, with PFAS. All these characteristics situate **1** as a promising candidate for PFAS decontamination.

### 2.2 PFAS Capture Experiments

Considering the mentioned characteristics of MOF **1**, we focused on studying its efficiency, as solid‐phase extraction (SPE) sorbent, toward a mixture containing a varied selection of representative long‐chain (≥C_7_) PFAS – such as, Perfluoroheptanoic acid (PFHpA), Perfluoroheptanesulfonic acid (PFHpS), Perfluorooctanoic acid (PFOA), Perfluorooctanesulfonic acid (PFOS), Perfluorononanoic acid (PFNA), Perfluorodecanoic acid (PFDA), Perfluoroundecanoic Acid (PFUnDA), Perfluorododecanoic acid (PFDoDA), 6:2 Fluorotelomer Sulfonic Acid (6‐2 FTSA), 8:2 Fluorotelomer Sulfonic Acid (8‐2 FTSA), 9‐chlorohexadecafluoro‐3‐oxanone‐1‐sulfonic acid (9Cl‐PF3ONS) and 11‐Chloroeicosafluoro‐3‐oxaundecane‐1‐sulfonic acid (11Cl‐PF3UdS)–, and of the highly elusive short‐chain (C_4_‐C_6_) PFAS –like Perfluorobutanoic acid (PFBA), Perfluorobutanesulfonic acid (PFBS), Perfluoropentanesulfonic acid (PFPeS), Perfluorohexanoic acid (PFHxA) and Perfluorohexanesulfonic acid (PFHxS). All analytical results are reported in **Table** **1** and Tables S2–S8 (Supporting Information).

**Table 1:** Comparison of removal efficiencies (%) of MOF **1** and powdered activated carbon (PAC) from an aqueous solution (pH 7.0) containing a mixture of selected PFAS (10 µg  L^−1^ for each compound). Measurements were carried out in triplicate. Relative Standard Deviation (RSD) in brackets.

PFAS	MOF 1 Removala^)^ (%RSD)	PAC Removala^)^ (%RSD)
PFBAb^)^	70 (3.5)	0 (0.0)
PFBSc^)^	86 (3.2)	1.1 (0.1)
PFPeSd^)^	99.7 (6.2)	41.7 (2.5)
PFHxAe^)^	78.2 (3.0)	10 (0.4)
PFHxSf^)^	100 (5.3)	77.7 (4.7)
PFHpAg^)^	96.7 (3.9)	39.4 (2.8)
PFHpSh^)^	100 (4.2)	97.6 (7.8)
PFOAi^)^	99.7 (5.6)	78.4 (3.9)
PFOSj^)^	100 (8.8)	100 (7.0)
PFNAk^)^	99.7 (8.0)	97.4 (5.8)
PFDAl^)^	100 (10.8)	100 (5.0)
PFUnDAm^)^	100 (11.0)	100 (5.0)
PFDoDAn^)^	100 (11.0)	100 (8.0)
6‐2 FTSAo^)^	99.5 (6.6)	92.5 (4.6)
8‐2 FTSAp^)^	100 (5.8)	100 (6.0)
9Cl‐PF3ONSq^)^	100 (6.0)	100 (4.0)
11Cl‐PF3UdSr^)^	100 (7.0)	100 (5.0)

^a)^ Expressed as percentage (%);

^b)^ PFBA = Perfluorobutanoic acid;

^c)^ PFBS = Perfluorobutanesulfonic acid;

^d)^ PFPeS = Perfluoropentanesulfonic acid;

^e)^ PFHxA = Perfluorohexanoic acid;

^f)^ PFHxS = Perfluorohexanesulfonic acid;

^g)^ PFHpA = Perfluoroheptanoic acid;

^h)^ PFHpS = Perfluoroheptanesulfonic acid

^i)^ PFOA = Perfluorooctanoic acid;

^j)^ PFOS = Perfluorooctanesulfonic acid;

^k)^ PFNA = Perfluorononanoic acid;

^l)^ PFDA = Perfluorodecanoic acid

^m)^ PFUnDA = Perfluoroundecanoic Acid;

^n)^ PFDoDA = Perfluorododecanoic acid;

^o)^ 6‐2 FTSA = 6:2 Fluorotelomer Sulfonic Acid;

^p)^ 8‐2 FTSA = 8:2 Fluorotelomer Sulfonic Acid;

^q^ 9Cl‐PF3ONS = 9‐chlorohexadecafluoro‐3‐oxanone‐1‐sulfonic acid;

^r)^ 11Cl‐PF3OUdS = 11‐Chloroeicosafluoro‐3‐oxaundecane‐1‐sulfonic acid.

SPE devices were prepared by packing 25 mg of MOF **1** between two frits within 1 mL empty polypropylene cartridges (Figure S1, Supporting Information). Capture experiments were conducted in triplicate (see tableheads of Tables 1; S3‐S6, Supporting Information), using 5 mL of an aqueous solution containing a commercial mixture of the selected PFAS at a concentration of 10 µg L^−1^ each, with the solution pH being 7.0. The contaminated solution was passed through the SPE device, and the quantification of the removed PFAS was performed *via* ultra‐high performance liquid chromatography–tandem mass spectrometry (UHPLC‐MS/MS) analysis of the percolated SPE fractions, which were filtered through a 0.22 µm polytetrafluoroethylene (PTFE) membrane (see Experimental Section, Table 1; Figure S1 and Tables S2‐S8, Supporting Information). In order to discard adsorption of PFAS on PTFE membrane, it was designed a blank experiment consisting on percolating a solution of known concentration of PFAS through only a PTFE membrane, which revealed no adsorption. Nevertheless, a test conducted with a cartridge containing frits but no MOF sorbent, to evaluate the non‐specific retention of PFAS on the support, revealed a slight uptake of certain long‐chain PFAS by the cartridge (Table S2, Supporting Information), at least during a single capture cycle. In this study, we have evaluated the removal efficiency of MOF **1** at 10 µg L^−1^, a concentration closer to environmentally relevant PFAS levels. Using these diluted conditions, rather than higher concentrations such as 1 ppm, allows for a more realistic assessment of the MOF's applicability in water remediation.

MOF **1** demonstrated removal efficiency of *ca*. 100% for all long‐chain PFAS in a single loading step (Table 1; Figure S2, Supporting Information) at pH = 7.0 in a low and relevant (ppb) concentration regime. Notably, unlike most adsorbents explored so far,^[^ ^13^, ^14^, ^15^, ^19^, ^21^, ^22^, ^40^, ^41^ ^]^ MOF **1** also achieved noticeable removal capacities for short‐chain PFAS (C_4_‐C_6_) such as PFBA, PFBS, PFPeS, PFHxA and PFHxS (70‐100%). Specifically, virtually 100% removal was observed for fourteen PFAS including both carboxylic and sulfonic acids like PFPeS (C_5_), PFHxS (C_6_), PFHpA (C_7_), PFHpS (C_7_), PFOA (C_8_), PFOS (C_8_), 9Cl‐PF3ONS (C_8_), PFNA (C_9_), 11Cl‐PF3OUdS (C_9_), PFDA (C_10_), 6‐2 FTSA (C_10_), 8‐2 FTSA (C_10_), PFUnDA (C_11_), and PFDoDA (C_12_). The three remaining PFAS –PFBA (C_4_), PFBS (C_4_) and PFHxA (C_6_)– were also effectively captured, with efficiencies of 70%, 86% and 78.2%, respectively. Indeed, the very good capture of short‐chain (C_4_‐C_6_) PFAS constitutes the most noteworthy result. This represents a remarkable advantage over other materials, such as activated carbons, zeolites or MOFs,^[^ ^13^, ^14^, ^15^, ^19^, ^21^, ^22^, ^40^, ^41^ ^]^ which have shown significant limitations in addressing these compounds. Moreover, these experiments were conducted with minimal PFAS−adsorbent contact time (<30 s), in a continuous‐flow SPE mode, highlighting the very fast adsorption kinetics of MOF **1**. These results place MOF **1** among the best adsorbents for capturing PFAS (particularly the short‐chain ones).

In order to validate the effectiveness of this material compared to traditional adsorbents, the efficiency of powdered activated carbon (PAC) in capturing these PFAS was also evaluated. Table 1 indeed shows the complete ineffectiveness of PAC in capturing short‐chain PFAS (raw UHPLC‐MS/MS spectra for each PFAS before and after adsorption process are provided in Figure S3, Supporting Information). Noteworthy, the capture experiments were also performed using a complex environmental matrix (pH = 7.3), such as real water from Turia river (Valencia, Spain). This allowed to reveal that the higher complexity of such waters, in terms of presence of natural organic matter and competitive ions, do not influence the PFAS adsorption on MOF **1**, as it shows similar results (Table S3, Supporting Information). However, more abrupt pH changes (to 4 and 10) do have a more significant impact on the capture efficiency, which is significantly reduced for short‐chain PFAS (Table S4, Supporting Information). The integrity of MOF **1** after capture experiments at pH = 4, 7 and 10 was ensured by measuring PXRD patterns (Figure S4, Supporting Information). Moreover, to further assess the water and pH stability of MOF **1**, the material was suspended in aqueous solutions at pH 4, 7, and 10 for 14 days. After this period, MOF **1** was recovered and analyzed by PXRD (Figure S5, Supporting Information top) and N_2_ adsorption isotherms (Figure S5, Supporting Information bottom), which displayed identical diffraction patterns and very similar adsorption capacities and Brunauer‐Emmett‐Teller^[^ ^61^ ^]^ (BET) surface areas (619.3, 694.6 and 635.4 m^2^/g, respectively). These results confirm the excellent stability of the framework in water, even under mildly acidic or basic conditions. Thus, considering that MOF **1** is not degraded at pH 4 and 10, the observed decrease in PFAS capture efficiency is likely related to changes in the charge state and interactions between PFAS and the MOF surface under non‐neutral conditions. At acidic pH (4), partial protonation of carboxylate PFAS reduces their anionic character and weakens electrostatic interactions, whereas at alkaline pH (10) hydroxide ions may compete for or coordinate to the metal sites of the MOF, altering the local charge distribution and blocking adsorption sites. In both cases, additional factors such as ion competition, modified solvation environments, or PFAS aggregation could further hinder adsorption, resulting in lower capture efficiency compared to the favorable balance of interactions observed at neutral pH.

Indeed, a review of the existing literature^[^ ^32^, ^33^, ^34^, ^36^, ^37^, ^38^, ^39^, ^40^, ^41^, ^42^, ^45^, ^46^, ^47^, ^48^, ^49^, ^50^, ^51^, ^52^, ^62^, ^63^ ^]^ on the application of MOFs for PFAS capture underscores their remarkable potential while also revealing substantial room for improvement. Early studies^[^ ^36^, ^37^, ^39^, ^45^, ^46^, ^47^, ^48^, ^49^ ^]^ primarily focused on examining host‐guest interactions, such as electrostatic, van der Waals, and hydrophobic interactions, which influence PFAS capture. However, these studies lacked quantitative assessments of the capture efficiency (%) of MOFs. More recent investigations^[^ ^38^, ^40^, ^41^, ^42^, ^43^, ^50^, ^51^, ^52^, ^62^, ^63^ ^]^ have confirmed the exceptional performance of certain MOFs in capturing long‐chain PFAS, with efficiencies ranging from 80% to 100%, albeit mostly limited to the widely studied PFOA and PFOS. Nevertheless, reported capture efficiencies for short‐chain PFAS^[^ ^40^, ^41^ ^]^ remained below 30%, further emphasizing the significance of the findings presented in this study.

### 2.3 Kinetics

**Figure 2:**
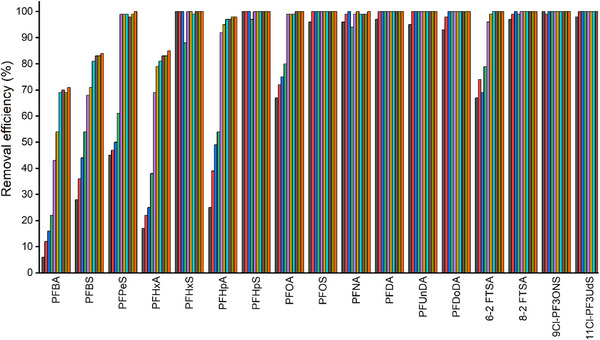
Removal efficiency (%) of MOF **1** for selected PFAS at different time intervals in dispersive mode. From left to right: 5 min. (gray), 15 min. (red), 30 min. (blue), 1 h. (green), 2 h. (purple), 4 h. (light orange), 6 h. (light blue), 8 h. (dark brown), 24 h. (light brown) and 30 h. (orange). Experimental data can be found in Table S5 (Supporting Information).

The continuous capture experiments carried out in this work go beyond to the common kinetic study in dispersive conditions –where the extraction is governed by equilibrium kinetics.^[^ ^64^ ^]^ However, in order to provide the most comprehensive study possible, we have also conducted a kinetic study for all the selected PFAS, by suspending MOF **1** in a solution containing all selected PFAS and measuring the removal efficiency at different time intervals ranging from 5 minutes to 30 hours. In this way, a moderately fast capture process is observed, and in the first measurement (5 minutes), 100% of many of these PFAS have already been captured (**Figure** **2**; Table S5, Supporting Information). Thus, due to the very rapid capture of most PFAS –precluding small retention at short times– and the above mentioned slight non‐specific retention of some long‐chain PFAS on the support (Table S2, Supporting Information), kinetic models could only be established for four compounds –PFBA, PFBS, PFHxA, and PFHpA– for which a pseudo‐first‐/second‐order model was determined (Figure S6, Supporting Information). As it can be seen from the tables for the pseudo‐first‐/second‐order models in this Figure, the adsorption data of tested analytes fitted best and perfectly well with the pseudo‐second‐order model (see R^2^ values).

### 2.4 Reusability

**Figure 3:**
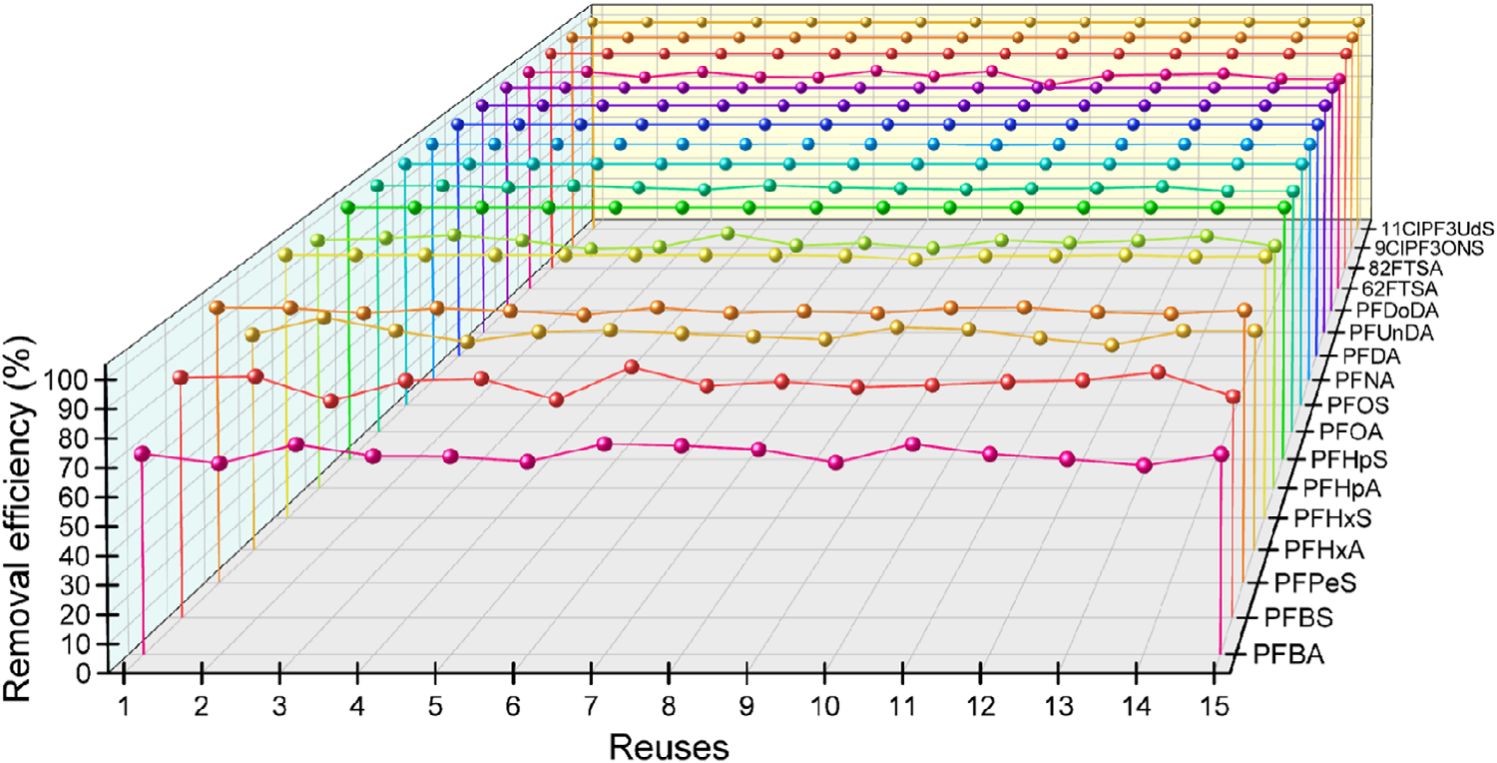
Reuses of MOF **1** toward the mixture containing the different seventeen PFAS. Experimental data can be found in Table S6 (Supporting Information).

Aiming at evaluating not only the effectiveness of the material but also its potential applicability in real‐world environments, the recyclability of the material was studied. In so doing, the capture efficiency of MOF **1** was assessed against an aqueous solution containing the 17 PFAS over 15 consecutive cycles. It was observed that the material is extraordinarily effective after all these reuse cycles, maintaining a 100% capture rate for the vast majority of these pollutants (**Figure** **3**; Table S6, Supporting Information). Moreover, no metal leaching was observed for MOF **1** during these capture experiments (confirmed by ICP‐MS analysis) and PXRD patterns for MOF **1**, after the 15th reuse cycle, show that **1** remains crystalline confirming its outstanding structural stability (Figure S7, Supporting Information).

The experimental procedure involved passing 10 µg L^−1^ aqueous samples containing the selected PFAS through the SPE cartridge and analyzing the eluents after each cycle. Notably, to assess the potential of MOF **1** for real‐world applications –where regenerating the material after each decontamination cycle is impractical– the cycles were carried out without full regeneration. Instead, only a small volume of eluent (5 mL of CH_3_OH followed by 5 mL of H_2_O) was passed through the cartridge between measurements to remove residual analytes adsorbed on the MOF particle surfaces and to recondition the cartridge.

### 2.5 Maximum Uptake Capacity

Aiming at elucidating the maximum sorption capacity of MOF **1** we carried out PFAS insertion experiments with four selected sulfur containing PFAS. Thus, polycrystalline samples of **1** (50 mg) were soaked in saturated water solutions of PFBS, PFPeS, PFHpS and PFOS for two weeks, replacing each saturated solution every 24 h. In so doing, maximum uptakes of 1221.2, 1397, 1108 and 1054.8 mg g^−1^ were determined, via elemental analysis, for **1**+PFBS, **1**+PFPeS, **1**+PFHpS and **1**+PFOS, respectively, which closely correspond to 2, 2, 1 and 1 PFAS molecules, respectively, per metal center (see Supporting Information for details). These results place MOF **1** among the materials with the highest adsorption capacity reported (Table S9, Supporting Information).^[^ ^32^, ^33^, ^34^, ^44^ ^]^ N_2_ adsorption isotherms for the adsorbates **1**+PFBS, **1**+PFPeS, **1**+PFHpS and **1**+PFOS (Figure S8, Supporting Information) show a significant decrease in adsorption properties in the mentioned adsorbates compared to pristine MOF **1**, further confirming that their pores are densely occupied by these guest molecules. Specifically, they exhibit BET surface areas of 673.7 (**1**), 28.9 (**1**+PFBS), 24.4 (**1**+PFPeS), 18.7 (**1**+PFHpS) and 17.3 (**1**+PFOS) m^2^/g, which closely correspond to that determined from the crystal structure of **1** and an abrupt decrease in the surface areas for the host‐guest adsorbates.

### 2.6 Single‐Crystal to Single‐Crystal Insertion Experiments

On the basis of the well‐known robustness and crystallinity of MOF **1** and aiming at unveiling the mechanisms governing such efficient capture properties reported, PFAS insertion experiments were carried out on single crystals of **1** (see Supporting Information, experimental section). In particular, suitable samples of host‐guest aggregates of **1** with PFBA and PFOS for single‐crystal X‐ray diffraction (SCXRD) could be obtained, and the crystal structure of PFBA@Cu^II^_2_(*S*,*S*)‐hismox (**PFBA@1**) and PFOS@Cu^II^_2_(*S*,*S*)‐hismox (**PFOS@1**) could be determined (Table S1, Supporting Information). Powder X‐Ray diffractions patterns (Figures S9 and S10, Supporting Information) confirm the purity homogeneity of the bulk sample, as they are identical to those theoretical patterns obtained from SCXRD.

**Figure 4:**
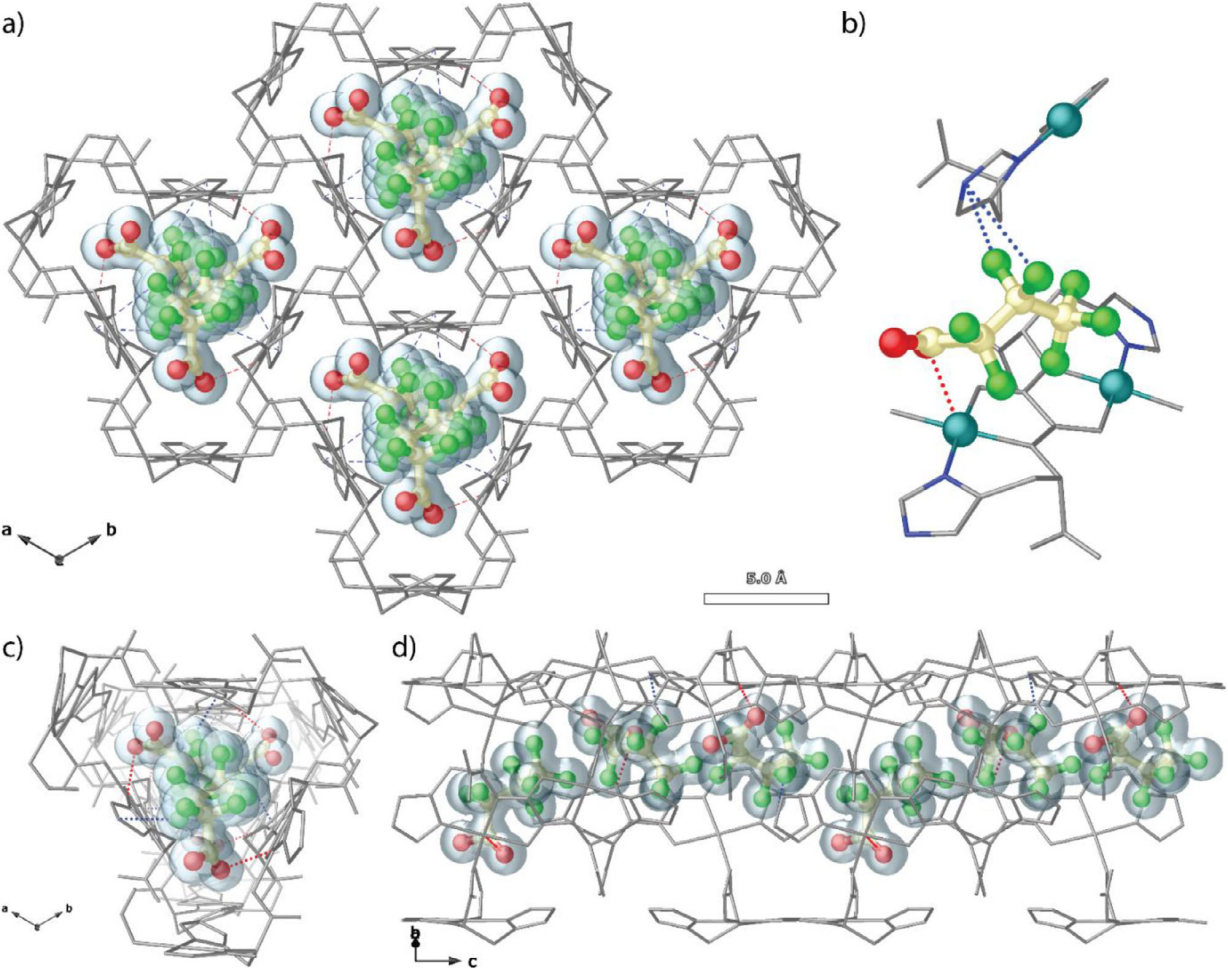
a) Perspective view of the 3D open‐framework of **PFBA@1** along the *c* axis. b) Fragment of **PFBA@1** emphasizing the interactions of PFBA with the framework. Single channel of **PFBA@1** along the *c* axis c) and the *bc* plane d). Cu atoms and ligands from the network are represented as grey sticks. F, C and O atoms from PFBA units are depicted as green, pale yellow and red spheres, respectively. PFBA molecules are highlighted with a blue surface in (a), (c) and (d).

**Figure 5:**
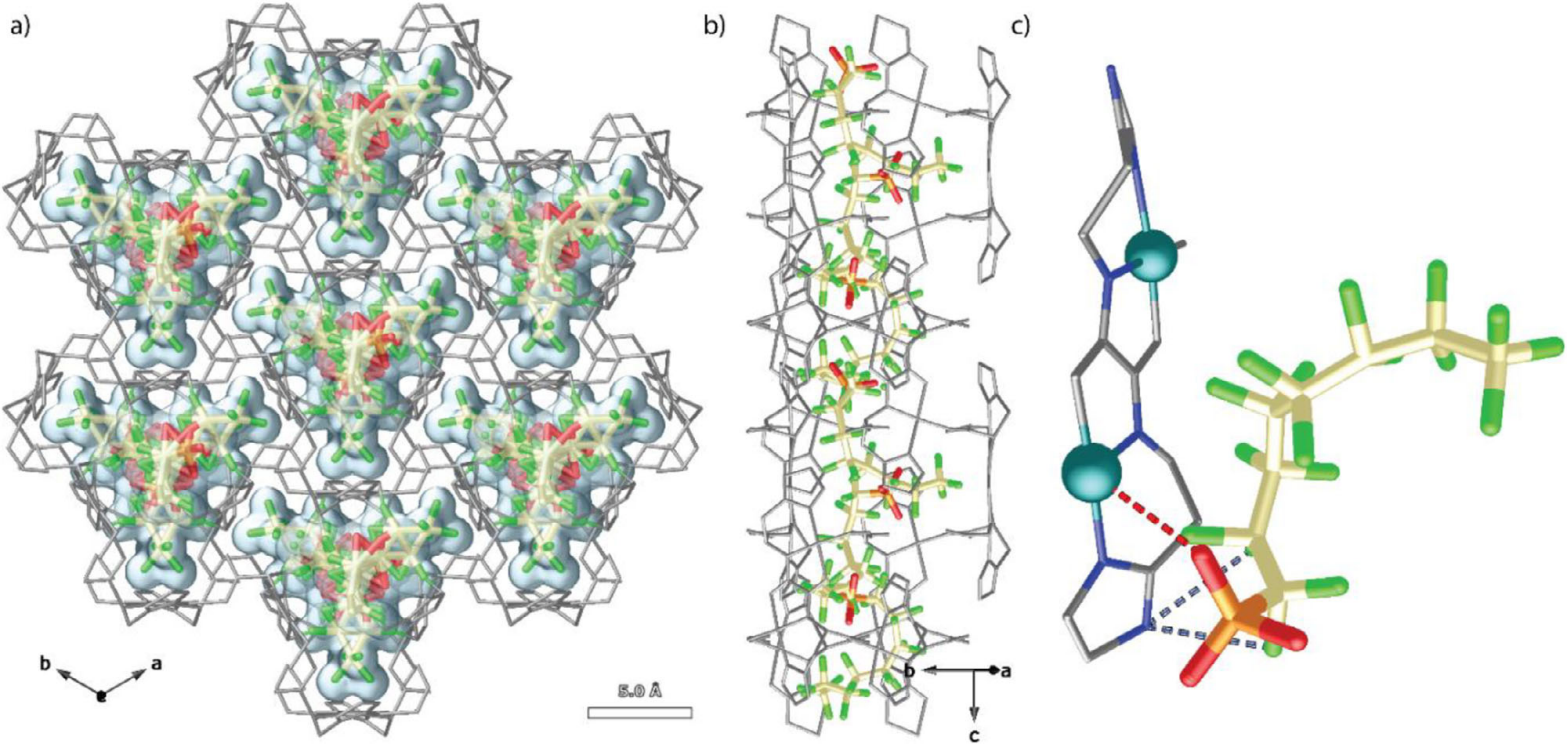
a) Perspective view of the 3D open‐framework of **PFOS@1** along the *c* axis. b) Single channel of **PFOS@1** along the *c* axis. c) Fragment of **PFOS@1** emphasizing the interactions of PFOS with the framework (red and blue dashed line). Metal atoms and ligands from the network are depicted as grey sticks with the exception of nitrogen atoms from imidazole rings and copper atoms which are presented by blue sticks and cyan spheres in (c). F, C and O atoms from PFBA units are depicted as green, pale yellow and red spheres, respectively. PFOS molecules are highlighted with a green surface in (a).

The as‐synthesized single crystals of **1** were immersed in aqueous solutions of PFBA and PFOS at room temperature for seven days. Subsequently, datasets for both **PFBA@1** and **PFOS@1** single crystals were collected at 300 K and 200 K, respectively, enabling the identification of the predominant locations and conformations of the PFBA and PFOS guest molecules. Compared to the hydrated pristine MOF, **PFBA@1** and **PFOS@1** remained isoreticular, with no appreciable lattice expansion (Table S1, Supporting Information). Crystallographically unique PFBA and PFOS molecules were identified within channels of **1** along the *c* axis (**Figures** **4** and **5**; Figures S11‐S15, Supporting Information).

PFBA coordinated with the open copper metal site via its carboxylate groups [Cu···O distance of 2.47 Å] (Figure S11, Supporting Information). Additional host‐guest interactions were primarily halogen bonds (C–F···N) with F···N separations ranging from 2.62 to 2.83 Å and C–F···π interactions with an F···centroid separation of 3.21 Å (Figure 4).

PFOS, on the other hand, coordinated with the open copper metal site via its sulfonic groups [Cu···O distance of 2.90 Å]. The crystal packing of PFOS molecules was further stabilized by halogen bonds, primarily C–F···N interactions (2.68–2.98 Å) and hydrogen bonds involving C–SO_3_···N interactions [O_2_S–O···N distances of 2.84 and 2.94 Å] (Figures 5; S12, Supporting Information). This additional interaction (hydrogen bonds), observed exclusively for PFOS, could be responsible for the slightly better removal efficiency of sulfonic acid–derived PFAS (see Table 1). Each Cu_2_(hismox) dimer binds to one PFBA molecule. Interestingly, slightly fewer PFOS molecules (0.67 per Cu_2_ dimer) were bound, likely due to the significant increase in molecular size from the C_4_ to the C_8_ chain. The remarkable adsorption behavior is undoubtedly supported by SCXRD analysis and can be attributed to the presence of unsaturated coordination sites in the pristine MOF **1**.

While the smaller PFBA molecules adopted a predominantly linear conformation (Figures S11 and S13, Supporting Information), the larger PFOS molecules were arranged differently within the pores, adopting a more bent conformation (Figure S12, Supporting Information). The sulfonic head group of PFOS was stabilized through coordination and supramolecular interactions, while the C8 tail remained more linear (Figures S12 and S14, Supporting Information). Crystallographically, this led to a largely disordered organization of PFOS, as described in the Supporting Information (SI). The spatial constraints imposed by the open coordination sites symmetries multiple the possible conformations of PFOS.

This is, as far as we know, the first time that coordinated PFBA and PFOS molecules have been directly identified through single‐crystal structure analysis. This highlights the crucial role of additional open coordination sites in the chemical adsorption of different PFAS molecules. Overall, both crystal structures (**PFBA@1** and **PFOS@1**) provide robust mechanistic insight into the high uptake capacities observed in bulk aqueous experiments. The conditions for bulk adsorption experiments and single‐crystal X‐ray diffraction (SCXRD) are necessarily different. Applying batch adsorption conditions is incompatible with SCXRD, as it would destroy the single‐crystal quality required for diffraction. Nonetheless, the SCXRD data provide an invaluable, high‐resolution snapshot of the binding mechanism by unequivocally identifying the host–guest interactions at the atomic level. The capture process is fundamentally driven by these supramolecular forces (coordination to copper, halogen bonding, etc.), and there is no physicochemical reason to assume they would be ineffective under batch conditions, where high adsorption is clearly observed.

## 3 Conclusion

In summary, this work demonstrates the exceptional performance of the bio‐derived MOF Cu^II^_2_(*S*,*S*)‐hismox · 5H_2_O (**1**) in the rapid and efficient capture of both short‐ and long‐chain PFAS from aqueous solutions. Thanks to its unique amphiphilic nanosized channels, accessible metal centers, and imidazole functionalities, MOF **1** exhibits remarkable affinity toward fourteen of seventeen widely used PFAS, achieving removal efficiencies up to 100%. In addition, highly elusive short‐chain PFAS, such as PFBA and PFBS, were removed with higher efficiency that in any reported material to date. The adsorption occurs with minimal contact time and shows excellent reusability, structural stability, and high uptake capacities. Moreover, single‐crystal X‐ray diffraction provided unprecedented structural insight into the binding modes of two representative short‐ and long‐chain PFAS within the MOF **1** framework, revealing coordination to copper centers and stabilizing supramolecular interactions. These findings situate MOF **1** as a highly promising and versatile platform for addressing one of the most pressing challenges in water remediation, offering a significant advancement over current technologies and laying the groundwork for further functional material design aimed at tackling persistent pollutants.

## Conflict of Interest

The authors declare no conflict of interest.

## Supporting information



Supporting Information

## Data Availability

The data that support the findings of this study are available in the supporting information of this article.</meta
