# Supplementary material for: Efficient Capture of Short‐ and Long‐Chain PFAS from Water by a Metal–Organic Framework
Source: Small. 2025 Nov 6;21(51):e10000. doi: 10.1002/smll.202510000 (PMC12723331; doi:10.1002/smll.202510000)
Supplement: Supplementary file 1 — Supporting Information [file SMLL-21-e10000-s001.pdf]

NANO · MICRO
small

Supporting Information

for *Small*, DOI 10.1002/smll.202510000

# Efficient Capture of Short- and Long-Chain PFAS from Water by a Metal–Organic Framework

*Thais Grancha, Patricia García-Atienza, Lidia García, Sergio Armenta*, José Manuel Herrero-Martínez, Donatella Armentano, Teresa F. Mastropietro, Jesús Ferrando Soria* and Emilio Pardo**

# Supporting Information

**Efficient Capture of Short- and Long-Chain PFAS from Water by a Metal-Organic Framework**

*Thais Grancha,*[†] *Patricia García-Atienza,*[†] *Lidia García, Sergio Armenta,*[*] *José Manuel Herrero-Martínez, Donatella Armentano, Teresa F. Mastropietro, Jesús Ferrando–Soria*[*] *and Emilio Pardo*[*]

Thais Grancha, Lidia García, Jesús Ferrando–Soria and Emilio Pardo
Instituto de Ciencia Molecular (ICMol), Universidad de Valencia, 46980 Paterna, Valencia, Spain
E-mail: jesus.ferrando@uv.es, emilio.pardo@uv.es

Patricia García-Atienza, Sergio Armenta and José Manuel Herrero-Martínez
Departamento de Química Analítica, Universitat de València, c/Dr. Moliner, 50, 46100 Burjassot, Valencia, Spain
E-mail: sergio.armenta@uv.es

Donatella Armentano and Teresa F. Mastropietro
*Dipartimento di Chimica e Tecnologie Chimiche (CTC), Università della Calabria, Rende 87036, Cosenza, Italy*

[†]These authors equally contributed to this work

## Experimental Section

**Materials.** All chemicals were of reagent grade quality. They were purchased from commercial sources and used as received.

**Physical Techniques:** Elemental (C, H, S, N) analyses were performed at the Microanalytical Service of the Universitat de València. FT–IR spectra were recorded on a Perkin-Elmer 882 spectrophotometer as KBr pellets.

The $N_2$ adsorption-desorption isotherms at 77 K were carried out on crystalline samples of **1**, **1**+PFBS, **1**+PFPeS, **1**+PFHpS and **1**+PFOS, with a Belsorp MINI X instrument. A sample of **1** was activated at 343 K under reduced pressure ($10^{-6}$ Torr) for 16 h prior to carrying out the sorption measurement. Samples of **1**+PFBS, **1**+PFPeS, **1**+PFHpS, and **1**+PFOS were recovered from the solid-phase extraction (SPE) device, and dried under vacuum at room temperature. Then, they were introduced into the adsorption sample cells, and activated at 343 K under reduced pressure ($10^{-6}$ Torr) for 16 h prior to carrying out the sorption measurements.

*Preparation of $Cu^{II}_2$(S,S)-hismox · $5H_2O$ (**1**).*

MOF **1** was synthesized following a previously reported procedure,[1] yielding both single crystals and polycrystalline powders. Well-defined deep blue cubic prisms suitable for single-crystal X-ray diffraction were obtained after several days by slow layer diffusion at room temperature in an assay tube. In the tube, a layer containing an aqueous solution of the precursor $(Bu_4N)_2\{Cu_2$(S,S)-hismox$_2\}\cdot 4H_2O$[1] (0.1 mmol in 2 mL of water) was placed first. Subsequently, an acidic aqueous solution (12 mL), adjusted to pH 2 with HCl, was carefully layered on top. The resulting crystals were collected by filtration and air-dried (0.045 g, 77% yield); elemental analysis calculated (%) for $C_{14}H_{22}Cu_2N_6O_{11}$ (577.5): C 29.12, H 3.84, N 14.55; found: C 28.87, H 3.69, N 14.57. IR (KBr) 3379 cm$^{-1}$ ($\nu_{N-H}$) and 1611 cm$^{-1}$ ($\nu_{C=O}$).

Alternatively, compound **1** can also be synthesized on a gram scale. For this, 2.5 g (2.32 mmol) of $(Bu_4N)_2\{Cu_2$(S,S)-hismox$_2\}\cdot 4H_2O$[1] were dissolved in 25 mL of water. The resulting dark green

solution was gradually acidified by the slow addition of an aqueous HCl solution (pH = 2) under continuous stirring, until reaching a final pH of approximately 4.0. The resulting blue polycrystalline solid was collected by filtration, washed with water and methanol, and dried under vacuum. Yield: 1.21 g, 91%; Anal.: calcd for $C_{14}H_{22}Cu_2N_6O_{11}$ (577.5): C 29.12, H 3.84, N 14.55; found: C 29.03, H 3.78, N 14.61. IR (KBr) 3364 $cm^{-1}$ ($\nu_{N-H}$) and 1602 $cm^{-1}$ ($\nu_{C=O}$).

*Syntheses of PFBA@Cu$^{II}_2$(S,S)-hismox (**PFBA@1**) and PFOS@Cu$^{II}_2$(S,S)-hismox (**PFOS@1**).*

Well-formed hexagonal green prisms of **PFBA@1** and **PFOS@1**, which were suitable for X-ray diffraction, were obtained by soaking crystals of **1** (*ca.* 5.0 mg) in aqueous solutions of PFBA and PFOS, respectively, for seven days. The water solutions were replaced every 12 h. The crystals were isolated by filtration on paper and air-dried. **PFBA@1:** Anal.: calcd for $C_{52.50}Cu_6H_{36}N_{18}O_{24}F_{16.50}$ (1997.73): C, 31.56; H, 1.82; N, 12.62%. Found: C, 31.65; H, 1.79; N, 12.59%. IR (KBr): $\nu$ = 1607 $cm^{-1}$ (C=O). **PFOS@1:** Anal.: calcd for $C_{58}Cu_6H_{36}N_{18}O_{24}F_{34}S_2$ (2460.41): C, 28.31; H, 1.48; S, 2.61; N, 10.25%. Found: C, 28.34; H, 1.39; S, 2.57; N, 10.11%. IR (KBr): $\nu$ = 1608 $cm^{-1}$ (C=O).

Maximum uptake capacities of **1**: The maximum uptake capacities of compound **1**, for each selected sulphur-containing PFAS, was determined. For that, *ca.* 50 mg of **1** were soaked in aqueous saturated solutions of the corresponding PFAS [PFBA, PFBS, PFOA and PFOS] during two weeks. Each saturated solution was replaced every 24 hours in order to ensure the maximum loading. After this period, each hybrid material was filtered off and gently washed with water. The PFAS contents of each material was determined by C, H, S, N analyses (*vide infra*).

C, H, N, S analyses: **1 + PFBS:** Anal.: calcd for $C_{22}Cu_2H_{14}S_2F_{18}N_6O_{12}$ (1087.58): C, 24.30; H, 1.30; S, 5.90; N, 7.73%. Found: C, 24.27; H, 1.35; S, 5.87; N, 7.77%. **1 + PFPeS:** Anal.: calcd for $C_{22.5}Cu_2H_{13.7}S_{1.7}F_{18.7}N_6O_{11.1}$ (1082.56): C, 24.96; H, 1.27; S, 5.03; N, 7.76%. Found: C, 24.99; H, 1.21; S, 5.02; N, 7.72%. **1 + PFHpS:** Anal.: calcd for $C_{22.4}Cu_2H_{13.2}S_{1.2}F_{18}N_6O_{9.6}$ (1027.52): C, 26.18; H, 1.29; S, 3.74; N, 8.18%. Found: C, 26.17; H, 1.27; S, 3.77; N, 8.23%. **1 + PFOS:** Anal.: calcd for

$C_{22}Cu_2H_{13}SF_{17}N_6O_9$ (987.5): C, 26.76; H, 1.33; S, 3.25; N, 8.51%. Found: C, 26.65; H, 1.29; S, 3.34; N, 8.49%.

**Analytical experiments:**

*- Reagents and materials:*

A multicomponent stock solution of 2 mg $L^{-1}$ concentration level in MeOH of perfluorobutanoic acid (PFBA), perfluorobutanesulfonic acid (PFBS), perfluoropentanesulfonic acid (PFPeS), perfluorohexanoic acid (PFHxA), perfluorohexanesulfonic acid (PFHxS), perfluoroheptanoic acid (PFHpA), perfluoroheptanesulfonic acid (PFHpS), perfluorooctanoic acid (PFOA), perfluorooctanoicsulfonic acid (PFOS), perfluorononanoic acid (PFNA), perfluorodecanoic acid (PFDA),perfluoroundecanoic acid (PFUnDA), perfluorododecanoic acid (PFDoDA), 6:2 fluorotelomer sulfonic acid (6-2 FTSA), 8:2 fluorotelomer sulfonic acid (8-2 FTSA), 9-Chlorohexadecafluoro-3-oxanone-1-sulfonic acid (9Cl-PF3ONS) and 11-Chloroeicosafluoro-3-oxaundecane-1-sulfonic acid (11Cl-PF3OUdS) was obtained from Phenomenex (Torrance, CA, USA). See details and structures in Scheme S1. All the solvents (*e.g.* methanol (MeOH) or acetonitrile (MeCN) and others) were all HPLC grade and purchased from VWR International Eurolab (Barcelona, Spain). Nanopure water was purified in Crystal B30 EDI Adrona deionizer (Riga, Latvia). Other non-specific reagents were of analytical grade unless otherwise stated. SPE propylene cartridges of 1 mL (internal volume) and their respective frits (1/16′, 20 μm) were provided from Análisis Vínicos (Tomelloso, Spain). Standard mixtures were prepared by dilution of this stock solution. Calibration curves ranging from 0.5 to 20 µg $L^{-1}$ were also prepared by diluting this multicomponent stock solution in water.

$^{13}C_2$-PFOA: Perfluorooctanoic Acid-13C2, $^{13}C_4$-PFOS: Perfluorooctanesulfonic Acid 13C4 (1,2,3,4-13C4) were used as isotope-labeled internal standards. Sodium$^{13}C_2$-PFOA (1 µg $mL^{-1}$) and $^{13}C_4$-PFOS (3 µg $mL^{-1}$) were provided by LGC standards. Internal standards were added to the PFAS solutions prior to the LC-MS/MS analysis at 5 µg $L^{-1}$ concentration level.

*- Instrumentation:*

For the solid-phase extraction (SPE) protocols, manifold (VacElut) with twelve positions (Agilent Technologies, Waldbronn, Germany,) and pump for vacuum N938 Laboport (KNF, Freiburg, Germany) were used. For UHPLC-tandem mass spectrometry (MS/MS) analysis, an AB SCIEX (Redwood City, CA, USA) ExionLC AD coupled to a Sciex QTRAP 6500+ mass spectrometer system was employed. Separations were performed in a C18 BEH chromatographic column (50 × 2.1 mm, 1.7 μm particle size) from Waters (Milford, Massachusetts, USA), and a 0.35 mL $min^{-1}$ mobile phase flow rate. The mobile phase was made up of 2 mM ammonium formate in water (A) and 2.5 mM ammonium formate in MeCN (B), using a gradient from 5 to 30% B for 0.5 min, from 30 to 95% B for 11.5 min, 95% B is maintaining for 8 min and from 95 to 5% B for 3 min. MS/MS acquisitions were done using electrospray ionization, with a source temperature of 360 $^{o}$C and an ion spray voltage of 4.5 kV. Data were evaluated using the PeakView™ software from AB SCIEX. To perform quantitation of analytes, multiple reaction monitoring (MRM) mode was used. Two MRM transitions were obtained for each compound (see Table S7).

*-Preparation of the cartridge:*

The MOF was packed in the SPE cartridge using a manual procedure. It involved the following steps:

1. Bottom frit loading. The bottom frit is pushed to the underside of the empty cartridge using a push rod.

2. Addition of the weighted mass of MOF. To do that, the cartridge is placed upright and the required amount of sorbent material is introduced in the cartridge. 3. Load the top frit by pushing the frit horizontally into the cartridge and securing the sorbent.

In all the cases, it has been assessed that no gaps appear between the top frit and the sorbent. If a gap appears, gently tap the cartridge wall to settle the sorbent and then push the top frit into its final position. Consistent sample and standards flow has been observed in the performed experiments.

*-Analytical features of the method:*

The developed method based on UHPLC-MS/MS after SPE extraction was validated in terms of working range, linearity, limit of detection (LOD) and quantification (LOQ), and precision (see Table S8). First of all, the matrix effect (ME) was evaluated through the comparison of the slope of an external calibration curve and the slope of a matrix-matched calibration curve (obtained by spiking the SPE extracts of a blank environmental water sample). As expected, there was no significant modulation in signal, either suppression or enhancement beyond the range of -20 to 20 % for all analytes. Thus, an external calibration line was proposed for analyte quantification.

Calibration curves were obtained for the analytes at concentration levels from the LOQ to 20 µg $L^{-1}$ and spiked with the internal standards (5 µg $L^{-1}$). The obtained calibration curves demonstrated appropriate linearity, as evidenced by the satisfactory coefficient of determination ($R^2$ > 0.9904) in all cases, as presented in Table S8, and by the back-calculated concentrations of the calibration standards, which met the acceptance criteria for residuals (within ± 15 % of the nominal value, except for the LOQ for which it should be within ± 20 %).

Precision, evaluated as relative standard deviation (% RSD), was obtained from replicate analyses of standard solutions across the working range (LOQ-20 µg $L^{-1}$) by performing 3 analytical runs. RSD values lower than 12 % were obtained in all cases, indicating good repeatability (Table S8).

The LOD and LOQ were estimated as 3 and 10 times, respectively, the standard deviation of the lowest concentration standard from the calibration lines (n=10) divided by the corresponding slope. As can be seen in Table S8, LOD and LOQ values ranged from 0.03 to 0.16 and 0.1 to 0.5 µg $L^{-1}$, respectively.

*-Removal experiments:*

As a starting point, the removal performance of MOF **1** was evaluated. To achieve this, SPE devices were prepared by placing 25 mg of MOF **1** between two frits inside 1 mL empty polypropylene cartridges. Capture experiments were conducted using an aqueous solution (pH = 7.0) containing a commercial mixture of the selected PFAS at a concentration of 10 µg $L^{-1}$. The SPE cartridge was conditioned using 3

mL methanol followed by 3 mL distilled water. After that, contaminated solution was passed through the SPE device at a flow rate of 1.5 mL min$^{-1}$. The solid sorbent was washed using 3 mL water and the retained compounds were eluted using 5 mL methanol. The quantification of the removed PFAS was determined by injecting the percolated SPE fractions (loading, washing and elution) —filtered through a PTFE membrane with a 0.22 µm pore size—into an LC-MS system.

*-Kinetic experiments:*

Kinetic experiments were performed by suspending 25 mg of MOF **1** in 10 mL sample solution containing all selected PFAS at a concentration of 10 µg L$^{-1}$ and measuring the removal efficiency at different time intervals ranging from 30 minutes to 30 hours. In order to do that, 50 µL of the sample solution was removed after each specific time, introduced in a chromatographic vial equipped with an insert and analyzed by the mentioned UHPLC-MS/MS procedure.

*-Reusability experiments:*

After PFAS capture, the solid sorbent is regenerated by passing 5 mL methanol at a flow rate of 1 mL min$^{-1}$ through the cartridge. After that, 5 mL water are passed to equilibrate the material before a new sample loading. PFAS concentration was measured in the sample loading that has passed through the cartridge and in the elution fraction.

*-PFAS quantification method with LC-MS/MS:*

The chromatographic analysis of the target compounds was performed using an UHPLC-MS/MS AB SCIEX ExionLC AD system coupled to a QTRAP 6500+ mass spectrometer from SCIEX (Darmstadt, Germany), featuring a quaternary pump for precise solvent delivery, a temperature-controlled autosampler maintained at 10 °C to ensure sample stability, and a column compartment regulated at 30 °C for optimal separation efficiency. Analyte separation was achieved using a C18 BEH reversed-phase column (50.0 x 2.1 mm, 1.7 µm particle size).

A 5 µL aliquot of each standard and sample extracts were introduced into the UHPLC system. The mobile phase consisted of (A) an aqueous solution with ammonium fluoride (2.5 mM) and (B) methanol with ammonium fluoride (2.5 mM), delivered at a flow rate of 0.35 mL $min^{-1}$. The elution gradient was programmed as follows: initial conditions of 5 % B, followed by a linear increase to 30 % B over 0.5 min, and then increased up to 95 % B for 12 min. This gradient composition was maintained during 8 min, and then returned to the initial conditions with a re-equilibration period of 5 min.

The triple quadrupole MS system operated in negative ESI mode, with the following optimized parameters: a spray voltage of -4.5 kV, a curtain gas of 35 psi, ionization gas and auxiliary gas pressure of 55 psi, and an ion source temperature of 360 °C. The specific precursor-to-product ion transitions monitored for each target analyte are detailed in Supplementary Table S7. A summary of the resulting relevant analytical parameters has been provided at Table S8.

*-Measurements with real samples:*

The environmental water sample, used to evaluate matrix effects, was passed through a solid-phase extraction (SPE) cartridge loaded with 25 mg of MOF **1**. The eluate obtained from this SPE column was subsequently used to prepare the matrix-matched calibration standards, maintaining the same water-to-methanol ratio as that employed in the adsorption experiments. These standards were analyzed by LC–MS and compared with conventional standards prepared by diluting the concentrated PFAS stock solution in LC–MS grade ultrapure water.

**X-ray crystallographic data collection and structure refinement:** Crystals of **PFBA@1**, and **PFOS@1** were selected and mounted on a MITIGEN holder in Paratone oil. They were measured at room temperature (**PFBA@1**) or T = 200 K (**PFOS@1**, under a nitrogen stream), to extract the best data set avoiding the possible amorphous contribution of disordered solvent lattice molecules. Diffraction data for both samples were acquired on a Bruker-Nonius X8APEXII CCD area detector diffractometer using graphite-monochromated Mo-Kα radiation (λ = 0.71073 Å), as a significant beam-damage was observed

for both single crystals under synchrotron radiation. Bearing in mind that crystal structure of adsorbates **PFBA@1**, and **PFOS@1** have been obtained measuring on crystals that suffered a single-crystal to single-crystal (SC to SC) process, it is reasonable. The data were processed through SAINT[2] reduction and SADABS[3] multi-scan absorption software. The structures were solved with the SHELXS structure solution program, using the Patterson method. The model was refined with version 2019/1 of SHELXL against $F^2$ on all data by full-matrix least squares.[4]

In both samples, all non-hydrogen atoms of the networks were refined anisotropically, whereas the highly dynamically disordered atoms of guest molecules were refined with restraints and constraints of rigid groups. For such kind of single crystals, the lower data quality for adsorbates, embedding highly disordered guest molecules, makes the use of constraints and especially restraints essential. The use of some bond lengths restraints applied on atoms belonging to highly dynamic moieties, has been reasonably imposed and related to the expected thermal motion, likely depending on the pore's size of the frameworks (FLAT, DFIX, DANG, SIMU, DELU and SADI) and several possible conformations of the guest molecules. In their refinements some further restraints, to make the refinement more efficient, have been applied. For instance, ADP components have been restrained to be like other related atoms, using SIMU for disordered sections or EADP for group of atoms of the guest molecules expected to have essentially similar ADPs. In the network of the **PFBA@1** MOF, all the hydrogen atoms of the networks were set in calculated position and refined isotropically using the riding model and hydrogen atoms on the guest molecules and on the solvent lattice molecules were neither found nor calculated. In fact, it is often expected that guest molecules are severely disordered, as a direct consequence of their high thermal motion and exhibited statistic disorder.

On the contrary, in **PFOS@1**, due to the impressive statistical disorder, other than dynamical one, hydrogen atoms where not defined neither for network nor for the guest molecules.

It should be also underlined that our best model has taken into account the most persistence conformations –within the additional complication– that even a low percentage, not taken into account,

such as a 10% of a whole PFAS guest molecule, is still a significant amount of electron density, and whole-molecule disorder lurks everywhere in the inky shadows of structure refinement, affecting quality of the model.

In **PFBA@1** and **PFOS@1** adsorbate's crystal structure refinements, the occupancies of the guests in the pores were fixed at 0.5 and 0.333, respectively in good agreement with bulk CHN analysis and size of the guest molecules. Unfortunately, also thermal motion has not been refined for any single disordered sites; it was refined with EADP for all the group of atoms of the guest molecules expected to have essentially similar ADPs. We strongly believe that it is the more reliable way to attempt to define loading instead of considering merely thermal factors, which can be affected by a lot of issues, above all severe disorder.

As stated above and as expected, in both adsorbates PFBA and PFOS guest molecules are statistically and thermally disordered. In **PFBA@1,** guest molecules exhibit two set of possible orientations of the guest molecules as detailed in Figure S14. The atom labelled C1P resides in special position, being shared by the two possible orientations of PFBA molecules in pores.

In **PFOS@1**, the statistical disorder, together with symmetry operations imposed by space group of the network and not allowed by guest molecules introduce further elements of large disorder. The partial overlap between different orientations at atom sites, makes the final refinement really challenging and to reach a suitable level of convergence it was not possible to refine hydrogen atoms.

Finally, in **PFBA@1**, and **PFOS@1** there are not free voids, in accordance with BET and SCXRD analysis (Figures S6 and S8-S9), indicating that the channels of **1** are entirely filled by PFAS molecules.

A summary of the crystallographic data and structure refinement for the two compounds is given in Table S1. The comments for the alerts A and B are reported in the CIFs using the validation response form (vrf). CCDC reference numbers are 2444172-2444173 for **PFBA@1** and **PFOS@1**, respectively.

The final geometrical calculations on free voids and the graphical manipulations were carried out with PLATON[5] implemented in WinGX,[6] and CRYSTAL MAKER[7] programs, respectively.

**X-ray Powder Diffraction Measurements:** Fresh polycrystalline samples of **1**, **1 after reuse experiments**, **PFBA@1** and **PFOS@1** were introduced into 0.5 mm borosilicate capillaries prior to being mounted and aligned on an Empyrean PANalytical powder diffractometer, using Cu Kα radiation ($\lambda$ = 1.54056 Å). For each sample, five repeated measurements were collected at room temperature ($2\theta$ = 2–60°) and merged in a single diffractogram.

**Table S1.** Summary of Crystallographic Data for **PFBA@1** and **PFOS@1**

| Compound | **PFBA@1** | **PFOS@1** |
|---|---|---|
| Formula | $C_{52.50}Cu_6H_{36}N_{18}O_{24}F_{16.50}$ | $C_{58}Cu_6H_{36}N_{18}O_{24}F_{34}S_2$ |
| $M$ (g mol$^{-1}$) | 1997.73 | 2460.41 |
| λ (Å) | 0.71073 | 0.71073 |
| Crystal system | hexagonal | hexagonal |
| Space group | $P3_121$ | $P3_121$ |
| $a$ (Å) | 10.489(4) | 10.5657(10) |
| $c$ (Å) | 16.760(4) | 16.787(2) |
| $V$ (Å$^3$) | 1597.0 (13) | 1623.0(4) |
| $Z$ | 3 | 1 |
| $\rho_{calc}$ (g cm$^{-3}$) | 2.077 | 2.517 |
| $\mu$ (mm$^{-1}$) | 2.110 | 2.201 |
| $T$ (K) | 300 | 200 |
| $\theta$ range for data collection (°) | 2.242 to 31.776 | 2.226 to 24.974 |
| Completeness to $\theta$ = 25.0 | 100% | 100% |
| Measured reflections | 15051 | 6159 |
| Unique reflections (Rint) | 3466 (0.2450) | 6159 (0.0695) |
| Observed reflections [$I > 2\sigma(I)$] | 1191 | 1587 |
| Goof | 1.134 | 1.360 |
| Absolute structure parameter (Flack) | 0.03(4) | 0.13(3) |
| $R^a$ [$I > 2\sigma(I)$] (all data) | 0.1058 (0.2669) | 0.1106 (0.1180) |
| $wR^b$ [$I > 2\sigma(I)$] (all data) | 0.2405 (0.2854) | 0.3036 (0.3129) |
| CCDC | 2444172 | 2444173 |

$^a$ $R = \sum(|F_o| - |F_c|)/\sum|F_o|$. $^b$ $wR = [\sum w(|F_o| - |F_c|)^2/\sum w|F_o|^2]^{1/2}$.

**Table S2.** Removal efficiencies (%) of the cartridge in the absence of MOF **1** / powdered activated carbon (PAC) using an aqueous contaminated solution containing a mixture of selected PFAS (10 μg $L^{-1}$ for each compound). Measurements were carried out in duplicate. Relative Standard Deviation (RSD) in brackets.

| PFAS | Cartridge Removal[a] (%RSD) |
|---|---|
| PFBA[a] | 2 (2) |
| PFBS[b] | 2 (3) |
| PFPeS[c] | 3 (4) |
| PFHxA[d] | 3 (4) |
| PFHxS[e] | 5 (4) |
| PFHpA[f] | 2 (3) |
| PFHpS[g] | 9 (5) |
| PFOA[h] | 8 (3) |
| PFOS[i] | 29 (11) |
| PFNA[j] | 12 (1) |
| PFDA[k] | 36 (11) |
| PFUnDA[l] | 50 (12) |
| PFDoDA[m] | 56 (11) |
| 6-2 FTSA[n] | 8 (4) |
| 8-2 FTSA[o] | 27 (8) |
| 9Cl-PF3ONS[p] | 45 (10) |
| 11Cl-PF3UdS[q] | 54 (8) |

[a]Expressed as percentage (%), [b]PFBA = Perfluorobutanoic acid, [c]PFBS = Perfluorobutanesulfonic acid, [d]PFPeS = Perfluoropentanesulfonic acid, [e]PFHxA = Perfluorohexanoic acid, [f]PFHxS = Perfluorohexanesulfonic acid, [g]PFHpA = Perfluoroheptanoic acid, [h]PFHpS = Perfluoroheptanesulfonic acid , [i]PFOA = Perfluorooctanoic acid , [j]PFOS = Perfluorooctanesulfonic acid, [k]PFNA = Perfluorononanoic acid , [l]PFDA = Perfluorodecanoic acid , [m]PFUnDA = Perfluoroundecanoic Acid, [n]PFDoDA = Perfluorododecanoic acid, [o]6-2 FTSA = 6:2 Fluorotelomer Sulfonic Acid, [p]8-2 FTSA = 8:2 Fluorotelomer Sulfonic Acid, [q]9Cl-PF3ONS = 9-chlorohexadecafluoro-3-oxanone-1-sulfonic acid, [r]11Cl-PF3OUdS = 11-Chloroeicosafluoro-3-oxaundecane-1-sulfonic acid.

**Table S3.** Comparison of removal efficiencies (%) of MOF **1** and powdered activated carbon (PAC) in a real environmental matrix –water from the Turia River (Valencia, Spain, pH = 7.3)– spiked with a mixture of selected PFAS (10 μg $L^{-1}$ for each compound). Measurements were carried out in triplicate. Relative Standard Deviation (RSD) in brackets.

| PFAS | MOF 1 Removal[a] (%RSD) | PAC Removal[a] (%RSD) |
|---|---|---|
| PFBA[a] | 72.9 (2.0) | 3 (0.2) |
| PFBS[b] | 89.1 (3.6) | 2.3 (0.1) |
| PFPeS[c] | 97 (5.4) | 34.1 (2.3) |
| PFHxA[d] | 83.1 (2.8) | 4.5 (0.5) |
| PFHxS[e] | 100 (5.0) | 77.4 (5.3) |
| PFHpA[f] | 100 (2.4) | 38 (3.0) |
| PFHpS[g] | 100 (3.8) | 100 (4.8) |
| PFOA[h] | 99.7 (6.2) | 84.2 (7.9) |
| PFOS[i] | 100 (4.4) | 93.9 (6.2) |
| PFNA[j] | 100 (2.1) | 98.2 (2.9)) |
| PFDA[k] | 100 (6.0) | 92 (4.0) |
| PFUnDA[l] | 100 (9.0) | 94.1 (4.9) |
| PFDoDA[m] | 100 (5.4) | 100 (7.0) |
| 6-2 FTSA[n] | 99.5 (6.5) | 92.5 (4.6) |
| 8-2 FTSA[o] | 98.7 (7.0) | 100 (3.8) |
| 9Cl-PF3ONS[p] | 100 (4.2) | 93.4 (3.0) |
| 11Cl-PF3UdS[q] | 100 (3.8) | 100 (4.2) |

[a]Expressed as percentage (%), [b]PFBA = Perfluorobutanoic acid, [c]PFBS = Perfluorobutanesulfonic acid, [d]PFPeS = Perfluoropentanesulfonic acid, [e]PFHxA = Perfluorohexanoic acid, [f]PFHxS = Perfluorohexanesulfonic acid, [g]PFHpA = Perfluoroheptanoic acid, [h]PFHpS = Perfluoroheptanesulfonic acid , [i]PFOA = Perfluorooctanoic acid , [j]PFOS = Perfluorooctanesulfonic acid, [k]PFNA = Perfluorononanoic acid , [l]PFDA = Perfluorodecanoic acid , [m]PFUnDA = Perfluoroundecanoic Acid, [n]PFDoDA = Perfluorododecanoic acid, [o]6-2 FTSA = 6:2 Fluorotelomer Sulfonic Acid, [p]8-2 FTSA = 8:2 Fluorotelomer Sulfonic Acid, [q]9Cl-PF3ONS = 9-chlorohexadecafluoro-3-oxanone-1-sulfonic acid, [r]11Cl-PF3OUdS = 11-Chloroeicosafluoro-3-oxaundecane-1-sulfonic acid.

**Table S4.** Removal efficiencies (%) of MOF **1** from an aqueous solution containing a mixture of selected PFAS (10 μg $L^{-1}$ for each compound) at pH = 4.0 (left) and pH = 10 (right). Measurements were carried out in triplicate. Relative Standard Deviation (RSD) in brackets.

| PFAS | Removal at pH = 4[a] (%RSD) | Removal at pH = 10[a] (%RSD) |
|---|---|---|
| PFBA[a] | 26 (2) | 28.8 (1.1) |
| PFBS[b] | 34 (5) | 35 (1.6) |
| PFPeS[c] | 64 (4) | 40 (3) |
| PFHxA[d] | 46 (3) | 40 (3) |
| PFHxS[e] | 62 (8) | 74 (5) |
| PFHpA[f] | 51 (3) | 52 (2) |
| PFHpS[g] | 84 (5) | 93 (4) |
| PFOA[h] | 60.6 (0.8) | 56 (6) |
| PFOS[i] | 97 (2) | 100 (0) |
| PFNA[j] | 89.8 (0.4) | 86 (5) |
| PFDA[k] | 98.7 (0.5) | 98.7 (0.7) |
| PFUnDA[l] | 100 (0) | 99.52 (0.17) |
| PFDoDA[m] | 100 (0) | 100 (0) |
| 6-2 FTSA[n] | 72.7 (0.4) | 65 (6) |
| 8-2 FTSA[o] | 99.1 (0.4) | 97.5 (0.9) |
| 9Cl-PF3ONS[p] | 99.6 (0.5) | 99.96 (0.04) |
| 11Cl-PF3UdS[q] | 100 (0) | 100 (0) |

[a]Expressed as percentage (%), [b]PFBA = Perfluorobutanoic acid, [c]PFBS = Perfluorobutanesulfonic acid, [d]PFPeS = Perfluoropentanesulfonic acid, [e]PFHxA = Perfluorohexanoic acid, [f]PFHxS = Perfluorohexanesulfonic acid, [g]PFHpA = Perfluoroheptanoic acid, [h]PFHpS = Perfluoroheptanesulfonic acid , [i]PFOA = Perfluorooctanoic acid , [j]PFOS = Perfluorooctanesulfonic acid, [k]PFNA = Perfluorononanoic acid , [l]PFDA = Perfluorodecanoic acid , [m]PFUnDA = Perfluoroundecanoic Acid, [n]PFDoDA = Perfluorododecanoic acid, [o]6-2 FTSA = 6:2 Fluorotelomer Sulfonic Acid, [p]8-2 FTSA = 8:2 Fluorotelomer Sulfonic Acid, [q]9Cl-PF3ONS = 9-chlorohexadecafluoro-3-oxanone-1-sulfonic acid, [r]11Cl-PF3OUdS = 11-Chloroeicosafluoro-3-oxaundecane-1-sulfonic acid.

**Table S5.** Removal efficiency of MOF **1** (%) for selected PFAS (using 10 μg $L^{-1}$ aqueous samples pH = 7.0) at different time intervals (5 min.-30h), in dispersive mode. Measurements were carried out in triplicate. Relative Standard Deviation (RSD) in brackets.

| **PFAS** | **Removal efficiency [a] at different time intervals (%RSD)** | | | | | | | | | |
|---|---|---|---|---|---|---|---|---|---|---|
| | **5 min.** | **15 min.** | **30 min.** | **1 h.** | **2 h.** | **4 h.** | **6 h.** | **8 h.** | **24 h.** | **30 h.** |
| PFBA[b] | 14 (3) | 13 (2) | 16 (1) | 22 (2) | 43 (4) | 54 (3) | 69 (5) | 70 (6) | 69 (6) | 71 (6) |
| PFBS[c] | 39 (1) | 47 (3) | 44 (4) | 54 (5) | 68 (6) | 71 (9) | 81 (9) | 83 (4) | 83 (3) | 84 (11) |
| PFPeS[d] | 45 (1) | 47 (4) | 50 (6) | 61 (4) | 99 (15) | 99 (11) | 99 (8) | 98 (7) | 99 (10) | 100 (7) |
| PFHxA[e] | 24 (7) | 29 (8) | 25 (3) | 38 (4) | 69 (3) | 79 (6) | 81 (6) | 83 (9) | 83 (8) | 85 (12) |
| PFHxS[f] | 100 (5) | 100 (4) | 100 (8) | 88 (5) | 100 (7) | 100 (15) | 99 (6) | 100 (8) | 100 (9) | 100 (5) |
| PFHpA[g] | 39 (4) | 47 (6) | 49 (4) | 54 (3) | 92 (12) | 95 (8) | 97 (5) | 97 (4) | 98 (9) | 98 (10) |
| PFHpS[h] | 100 (14) | 100 (14) | 100 (14) | 97 (5) | 100 (6) | 100 (7) | 100 (11) | 100 (14) | 100 (15) | 100 (9) |
| PFOA[i] | 67 (3) | 72 (4) | 75 (11) | 80 (6) | 99 (15) | 99 (12) | 99 (11) | 100 (8) | 100 (14) | 100 (12) |
| PFOS[j] | 96 (15) | 100 (15) | 100 (5) | 99 (5) | 100 (9) | 100 (11) | 100 (8) | 100 (5) | 100 (10) | 100 (13) |
| PFNA[k] | 96 (8) | 99 (3) | 100 (4) | 94 (4) | 99 (6) | 100 (4) | 99 (11) | 99 (11) | 99 (12) | 100 (13) |
| PFDA[l] | 97 (3) | 100 (10) | 100 (10) | 100 (7) | 100 (5) | 100 (6) | 100 (11) | 100 (8) | 100 (9) | 100 (13) |
| PFUnDA[m] | 95 (3) | 100 (2) | 100 (8) | 100 (14) | 100 (11) | 100 (12) | 100 (13) | 100 (9) | 100 (12) | 100 (6) |
| PFDoDA[n] | 93 (5) | 98 (7) | 100 (6) | 100 (12) | 100 (11) | 100 (9) | 100 (6) | 100 (15) | 100 (7) | 100 (13) |
| 6-2 FTSA[o] | 67 (1) | 74 (6) | 69 (8) | 79 (10) | 96 (6) | 99 (9) | 100 (4) | 100 (7) | 100 (4) | 100 (11) |
| 8-2 FTSA[p] | 97 (4) | 99 (6) | 100 (5) | 99 (15) | 100 (9) | 100 (10) | 100 (5) | 100 (8) | 100 (7) | 100 (6) |
| 9Cl-PF3ONS[q] | 100 (5) | 99 (7) | 100 (6) | 100 (4) | 100 (11) | 100 (11) | 100 (4) | 100 (11) | 100 (4) | 100 (6) |
| 11Cl-PF3UdS[r] | 98 (7) | 100 (8) | 100 (3) | 100 (12) | 100 (6) | 100 (6) | 100 (10) | 100 (8) | 100 (5) | 100 (9) |

[a]Expressed as percentage (%), [b]PFBA = Perfluorobutanoic acid, [c]PFBS = Perfluorobutanesulfonic acid, [d]PFPeS = Perfluoropentanesulfonic acid, [e]PFHxA = Perfluorohexanoic acid, [f]PFHxS = Perfluorohexanesulfonic acid, [g]PFHpA = Perfluoroheptanoic acid, [h]PFHpS = Perfluoroheptanesulfonic acid , [i]PFOA = Perfluorooctanoic acid , [j]PFOS = Perfluorooctanesulfonic acid, [k]PFNA = Perfluorononanoic acid , [l]PFDA = Perfluorodecanoic acid , [m]PFUnDA = Perfluoroundecanoic Acid, [n]PFDoDA = Perfluorododecanoic acid, [o]6-2 FTSA = 6:2 Fluorotelomer Sulfonic Acid, [p]8-2 FTSA = 8:2 Fluorotelomer Sulfonic Acid, [q]9Cl-PF3ONS = 9-chlorohexadecafluoro-3-oxanone-1-sulfonic acid, [r]11Cl-PF3OUdS = 11-Chloroeicosafluoro-3-oxaundecane-1-sulfonic acid.

**Table S6.** Removal efficiency (%) of the 17 selected PFAS by MOF **1**, for 15 consecutive cycles, using 10 μg $L^{-1}$ aqueous samples. Measurements were carried out in triplicate. Relative Standard Deviation (RSD) in brackets.

| **PFAS** | **Removal efficiencies[a] for 15 consecutive cycles (%RSD)** | | | | | | | | | | | | | | |
|---|---|---|---|---|---|---|---|---|---|---|---|---|---|---|---|
| | **1** | **2** | **3** | **4** | **5** | **6** | **7** | **8** | **9** | **10** | **11** | **12** | **13** | **14** | **15** |
| PFBA[b] | 69.2 (5.2) | 65.9 (4.9) | 72.4 (8.0) | 68.4 (5.5) | 68.3 (6.9) | 66.5 (6.7) | 72.6 (5.8) | 71.9 (5.6) | 70.7 (3.6) | 66.2 (2.8) | 72.5 (3.7) | 69.1 (4.4) | 67.4 (5.8) | 65.2 (6.1) | 69.1 (2.0) |
| PFBS[c] | 85 (5.1) | 85.4 (6.0) | 76.7 (7.7) | 83.9 (5.0) | 84.6 (8.5) | 77.1 (6.9) | 88.7 (8.0) | 82 (7.8) | 83.6 (4.2) | 81.6 (7.9) | 82.3 (2.9) | 83.5 (5.9) | 84.7 (2.4) | 86.9 (7.4) | 78.3 (2.1) |
| PFPeS[d] | 99.8 (6.0) | 99.7 (8.0) | 97.7 (3.9) | 99.6 (8.0) | 98.6 (5.9) | 97.1 (8.6) | 99.9 (4.0) | 97.8 (10.8) | 98.5 (4.8) | 97.7 (9.2) | 99.7 (8.6) | 99.9 (8.6) | 98.1 (7.9) | 97.5 (5.6) | 98.9 (7.4) |
| PFHxA[e] | 79.9 (7.8) | 86.2 (7.8) | 81.3 (3.3) | 77.3 (8.5) | 80.9 (7.6) | 81.6 (9.0) | 80.3 (6.4) | 79.2 (6.7) | 78.2 (8.4) | 82.6 (5.2) | 81.9 (3.8) | 78.6 (3.9) | 76 (7.3) | 81.3 (4.9) | 81.3 (6.7) |
| PFHxS[f] | 100 (9.0) | 100 (9.0) | 100 (7.0) | 100 (11.0) | 100 (9.0) | 100 (4.0) | 100 (7.0) | 100 (4.0) | 99.7 (11.0) | 98.2 (9.8) | 99.7 (8.0) | 99.7 (7.0) | 100 (9.8) | 99.4 (8.9) | 99.5 (4.0) |
| PFHpA[g] | 96.2 (9.6) | 97.1 (6.8) | 98.3 (8.8) | 96.2 (10.6) | 92.8 (4.6) | 93.7 (10.3) | 99 (6.9) | 94.3 (8.0) | 95.2 (6.0) | 93.2 (7.5) | 96.3 (7.1) | 95.3 (8.2) | 96.1 (6.5) | 97.9 (9.0) | 94.1 (4.9) |
| PFHpS[h] | 100 (9.0) | 100 (4.0) | 100 (8.0) | 100 (9.0) | 100 (8.0) | 100 (7.0) | 100 (8.0) | 100 (5.0) | 100 (5.0) | 100 (5.0) | 100 (6.0) | 100 (9.0) | 100 (5.0) | 100 (10.0) | 100 (9.0) |
| PFOA[i] | 99.8 (6.0) | 99.8 (10.0) | 99.1 (4.0) | 99.8 (8.0) | 99 (5.0) | 98.3 (5.9) | 99.9 (8.0) | 99.1 (5.9) | 98.7 (5.9) | 98.4 (3.9) | 98.7 (7.9) | 98.8 (8.9) | 99.4 (4.0) | 97.7 (10.8) | 97.7 (7.8) |
| PFOS[j] | 100 (6.0) | 100 (7.0) | 100 (11.0) | 100 (4.0) | 100 (8.0) | 100 (4.0) | 100 (9.0) | 100 (6.0) | 100 (5.0) | 100 (9.0) | 100 (6.0) | 100 (7.0) | 100 (8.0) | 100 (7.0) | 100 (5.0) |
| PFNA[k] | 99.8 (10.0) | 99.8 (11.0) | 99.8 (11.0) | 99.8 (10.0) | 99.8 (4.0) | 99.7 (9.0) | 99.9 (9.0) | 99.8 (9.0) | 99.8 (8.0) | 99.4 (8.0) | 99.7 (4.0) | 99.7 (4.0) | 99.8 (4.0) | 99.6 (10.0) | 99.7 (5.0) |
| PFDA[l] | 100 (6.0) | 100 (7.0) | 100 (11.0) | 100 (9.0) | 100 (8.0) | 100 (10.0) | 100 (9.0) | 100 (11.0) | 100 (4.0) | 100 (7.0) | 100 (5.0) | 100 (7.0) | 100 (7.0) | 100 (4.0) | 100 (5.0) |
| PFUnDA[m] | 100 (5.3) | 100 (6.0) | 100 (8.0) | 100 (7.2) | 100 (8.0) | 100 (7.0) | 100 (6.4) | 100 (8.0) | 100 (8.0) | 100 (8.0) | 100 (7.0) | 100 (9.0) | 100 (5.0) | 100 (6.7) | 100 (4.0) |
| PFDoDA[n] | 100 (8.0) | 100 (5.0) | 100 (8.0) | 100 (9.0) | 100 (4.0) | 100 (7.0) | 100 (8.0) | 100 (6.0) | 100 (10.0) | 100 (8.0) | 100 (6.0) | 100 (6.0) | 100 (9.0) | 100 (6.0) | 100 (80) |
| 6-2 FTSA[o] | 99 (8.9) | 99.3 (5.0) | 96.8 (5.8) | 99.2 (10.9) | 96.9 (7.8) | 96.7 (5.8) | 99.8 (8.0) | 97.3 (8.8) | 99.6 (10.0) | 93.3 (4.7) | 97.6 (8.8) | 98 (8.8) | 98.4 (9.8) | 96 (8.6) | 95.9 (9.6) |
| 8-2 FTSA[p] | 100 (7.0) | 100 (8.0) | 100 (6.0) | 100 (4.0) | 100 (9.0) | 100 (7.0) | 100 (7.0) | 100 (9.0) | 100 (8.0) | 100 (9.0) | 100 (9.0) | 100 (7.0) | 100 (6.0) | 100 (4.0) | 100 (8.0) |
| 9Cl-PF3ONS[q] | 100 (10.0) | 100 (5.0) | 100 (10.0) | 100 (6.0) | 100 (8.0) | 100 (9.0) | 100 (9.0) | 100 (6.0) | 100 (11.0) | 100 (4.0) | 100 (8.0) | 100 (6.0) | 100 (4.0) | 100 (11.0) | 100 (8.0) |
| 11Cl-PF3UdS[r] | 100 (9.0) | 100 (6.0) | 100 (10.0) | 100 (7.0) | 100 (8.0) | 100 (4.0) | 100 (7.0) | 100 (10.0) | 100 (8.0) | 100 (8.0) | 100 (4.0) | 100 (4.0) | 100 (6.0) | 100 (5.0) | 100 (5.9) |

[a]Expressed as percentage (%), [b]PFBA = Perfluorobutanoic acid, [c]PFBS = Perfluorobutanesulfonic acid, [d]PFPeS = Perfluoropentanesulfonic acid, [e]PFHxA = Perfluorohexanoic acid, [f]PFHxS = Perfluorohexanesulfonic acid, [g]PFHpA = Perfluoroheptanoic acid, [h]PFHpS = Perfluoroheptanesulfonic acid , [i]PFOA = Perfluorooctanoic acid , [j]PFOS = Perfluorooctanesulfonic acid, [k]PFNA = Perfluorononanoic acid , [l]PFDA = Perfluorodecanoic acid , [m]PFUnDA = Perfluoroundecanoic Acid, [n]PFDoDA = Perfluorododecanoic acid, [o]6-2 FTSA = 6:2 Fluorotelomer Sulfonic Acid, [p]8-2 FTSA = 8:2 Fluorotelomer Sulfonic Acid, [q]9Cl-PF3ONS = 9-chlorohexadecafluoro-3-oxanone-1-sulfonic acid, [r]11Cl-PF3OUdS = 11-Chloroeicosafluoro-3-oxaundecane-1-sulfonic acid.

**Table S7.** Summary of MRM transitions for the determination of the different PFAS under study.

| Analyte | $t_R$ (min) | Q1 (m/z) | Q3 quantifier (m/z) | Q3 qualifier (m/z) |
|---|---|---|---|---|
| **PFBA** | 8.6 | 213 | 169 | - |
| **PFBS** | 12.7 | 299 | 99 | 80 |
| **PFPeS** | 13.3 | 349 | 80 | 90 |
| **PFHxA** | 13.2 | 313 | 269 | 119 |
| **PFHxS** | 13.6 | 399 | 99 | 80 |
| **PFHpA** | 13.6 | 363 | 319 | 169 |
| **PFHpS** | 13.9 | 449 | 99 | 80 |
| **PFOA** | 13.9 | 413 | 369 | 169 |
| **PFOS** | 14.1 | 499 | 99 | 80 |
| **PFNA** | 14.1 | 463 | 419 | 219 |
| **PFDA** | 14.3 | 513 | 469 | 269 |
| **PFUnDA** | 14.5 | 563 | 519 | 269 |
| **PFDoDA** | 14.8 | 613 | 569 | 269 |
| **6-2 FTSA** | 13.9 | 427 | 407 | 81 |
| **8-2 FTSA** | 14.3 | 527 | 507 | 81 |
| **9Cl-PF3ONS** | 14.1 | 531 | 351 | - |
| **11Cl-PF3OUdS** | 14.6 | 631 | 451 | - |
| **$^{13}C_2$-PFOA** | 13.9 | 415 | 371 | - |
| **$^{13}C_4$-PFOS** | 14.1 | 503 | 80 | - |

$^{13}C_2$-PFOA: Perfluorooctanoic Acid-13C2.

$^{13}C_4$-PFOS: Perfluorooctanesulfonic Acid 13C4 (1,2,3,4-13C4) Sodium

$^{13}C_2$-PFOA (1 µg mL$^{-1}$) and $^{13}C_4$-PFOS (3 µg mL$^{-1}$) provided by LGC standards.
Internal satandards were added to the PFAS solutions prior to the LC-MS/MS analysis at 5 µg L$^{-1}$ concentration level.

**Table S8.** Summary of relevant analytical parameters.

| Analyte | Working range (µg $L^{-1}$) | Linearity ($R^2$) | LOD (µg $L^{-1}$) | LOQ (µg $L^{-1}$) | RSD (%) | Matrix effect (ME, %) |
|---|---|---|---|---|---|---|
| **PFBA** | 0.5-20 | 0.9997 | 0.09 | 0.3 | 6 | 4 |
| **PFBS** | 0.5-20 | 0.9908 | 0.16 | 0.5 | 6 | 9 |
| **PFPeS** | 0.5-20 | 0.9979 | 0.06 | 0.2 | 5 | 7 |
| **PFHxA** | 0.5-20 | 0.9987 | 0.06 | 0.2 | 8 | 6 |
| **PFHxS** | 0.5-20 | 0.9984 | 0.03 | 0.1 | 6 | 2 |
| **PFHpA** | 0.5-20 | 0.9963 | 0.06 | 0.2 | 11 | 2 |
| **PFHpS** | 0.5-20 | 0.9983 | 0.12 | 0.4 | 9 | 6 |
| **PFOA** | 0.5-20 | 0.9934 | 0.09 | 0.3 | 7 | 3 |
| **PFOS** | 0.5-20 | 0.9936 | 0.16 | 0.5 | 6 | 7 |
| **PFNA** | 0.5-20 | 0.9948 | 0.06 | 0.2 | 9 | 9 |
| **PFDA** | 0.5-20 | 0.9993 | 0.09 | 0.3 | 12 | 8 |
| **PFUnDA** | 0.5-20 | 0.9991 | 0.06 | 0.2 | 5 | 8 |
| **PFDoDA** | 0.5-20 | 0.9927 | 0.03 | 0.1 | 7 | 1 |
| **6-2 FTSA** | 0.5-20 | 0.9965 | 0.09 | 0.3 | 9 | 3 |
| **8-2 FTSA** | 0.5-20 | 0.9940 | 0.06 | 0.2 | 7 | 7 |
| **9Cl-PF3ONS** | 0.5-20 | 0.9904 | 0.09 | 0.3 | 5 | 4 |
| **11Cl-PF3OUdS** | 0.5-20 | 0.9961 | 0.16 | 0.5 | 10 | 8 |

**Table S9.** Summary of most relevant adsorbents found in the literature with reported adsorption capacities.

| MOF | PFAS | Adsorption Capacity (mg/g) | Reference |
|---|---|---|---|
| MIL-101(Cr) (PAM) | PFOA | 493 | 36 |
| MIL-101(Cr) (PSM) | PFOA | 783 | 36 |
| ZIF-L | PFOA | 497 | 37 |
| PCN-999 | PFOA | 1089 | 38 |
| NU-1000 | PFCAs | 201–604 | 41 |
| NU-1000 | PFSAs | 400–620 | 41 |
| MOF-808 | PFOA | 1581 | 42 |
| TFA-MOF-808 | PFOA | 2496 | 42 |
| DUT-67 | PFOA | 383 | 42 |
| TFA-DUT-67 | PFOA | 490 | 42 |
| FBA-DUT-67 | PFOA | 413 | 42 |
| DFBA-DUT-67 | PFOA | 405 | 42 |
| SCU-8 | PFOS | 44.79 | 45 |
| UiO-66-(F4) | PFOA | 467 | 46 |
| UiO-66-(F4) | PFOS | 254 | 46 |
| Defective UiO-66 | PFOS | 620 | 48 |
| Fe-BTC | PFOA | 418 | 49 |
| MIL-100(Fe) | PFOA | 349 | 49 |
| MIL-101(Fe) | PFOA | 370 | 49 |
| Magnetic COF | PFOA | 631 | 17 |
| PAC | PFOA | 175–524 | 16 |
| GAC | PFOA | 112–161 | 16 |
| SWCNT | PFOA | 79 | 18 |
| Chitosan-Molecular Imprinted Polymer | PFOS | 1455 | 23 |
| Anion-exchange resin | PFOA | 1168 | 20 |

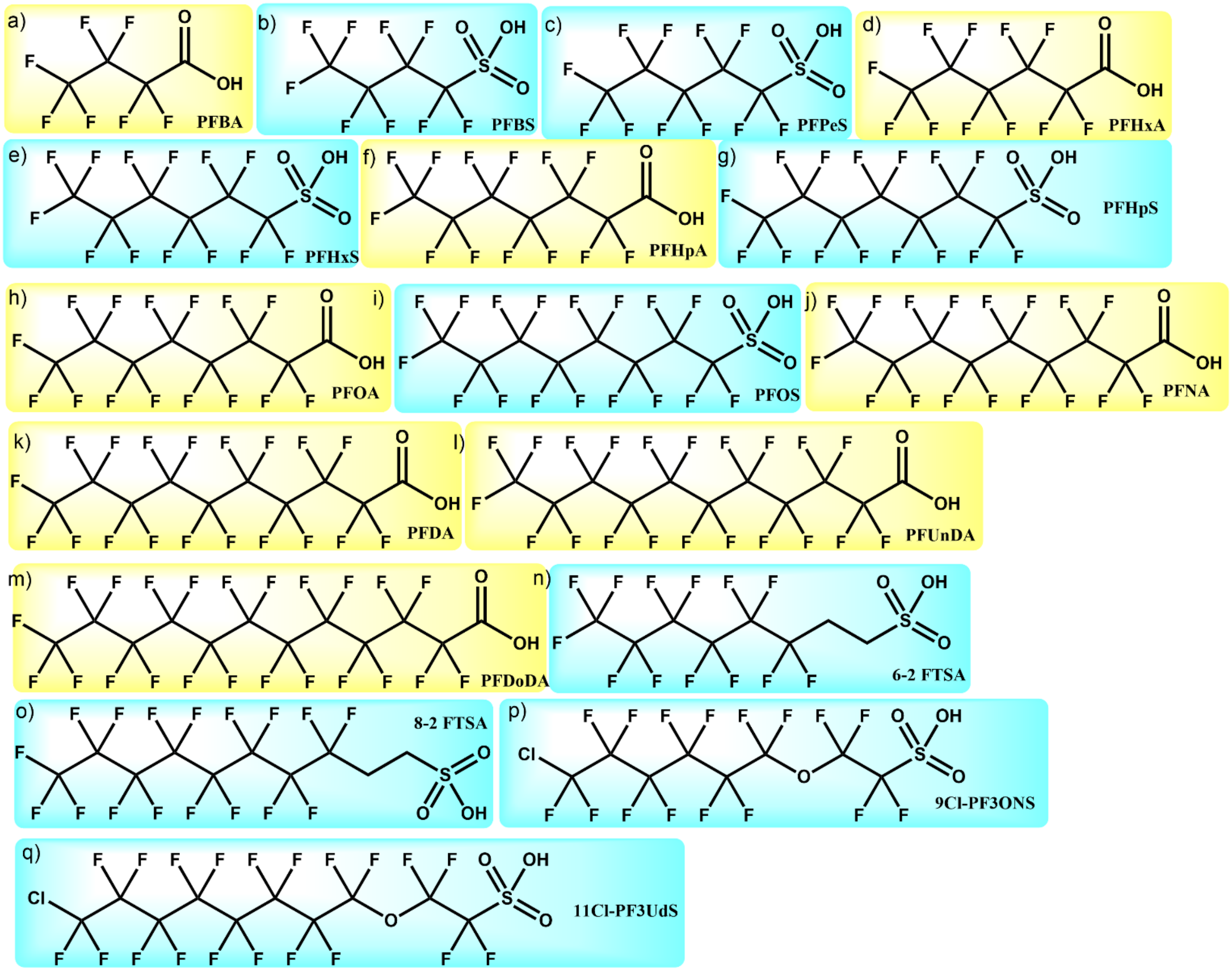


**Scheme S1**. Chemical structures of PFBA = Perfluorobutanoic acid (a), PFBS = Perfluorobutanesulfonic acid (b), PFPeS = Perfluoropentanesulfonic acid (c), PFHxA = Perfluorohexanoic acid (d), PFHxS = Perfluorohexanesulfonic acid (e), PFHpA = Perfluoroheptanoic acid (f), PFHpS = Perfluoroheptanesulfonic acid (g), PFOA = Perfluorooctanoic acid (h), PFOS = Perfluorooctanesulfonic acid (i), PFNA = Perfluorononanoic acid (j), PFDA = Perfluorodecanoic acid (k), PFUnDA = Perfluoroundecanoic Acid (l), PFDoDA = Perfluorododecanoic acid (m), 6-2 FTSA = 6:2 Fluorotelomer Sulfonic Acid (n), 8-2 FTSA = 8:2 Fluorotelomer Sulfonic Acid (o), 9Cl-PF3ONS = 9-chlorohexadecafluoro-3-oxanone-1-sulfonic acid (p), [q]11Cl-PF3OUdS = 11-Chloroeicosafluoro-3-oxaundecane-1-sulfonic acid (q). Sulfonic and carboxylic acids are highlighted in blue and yellow, respectively.

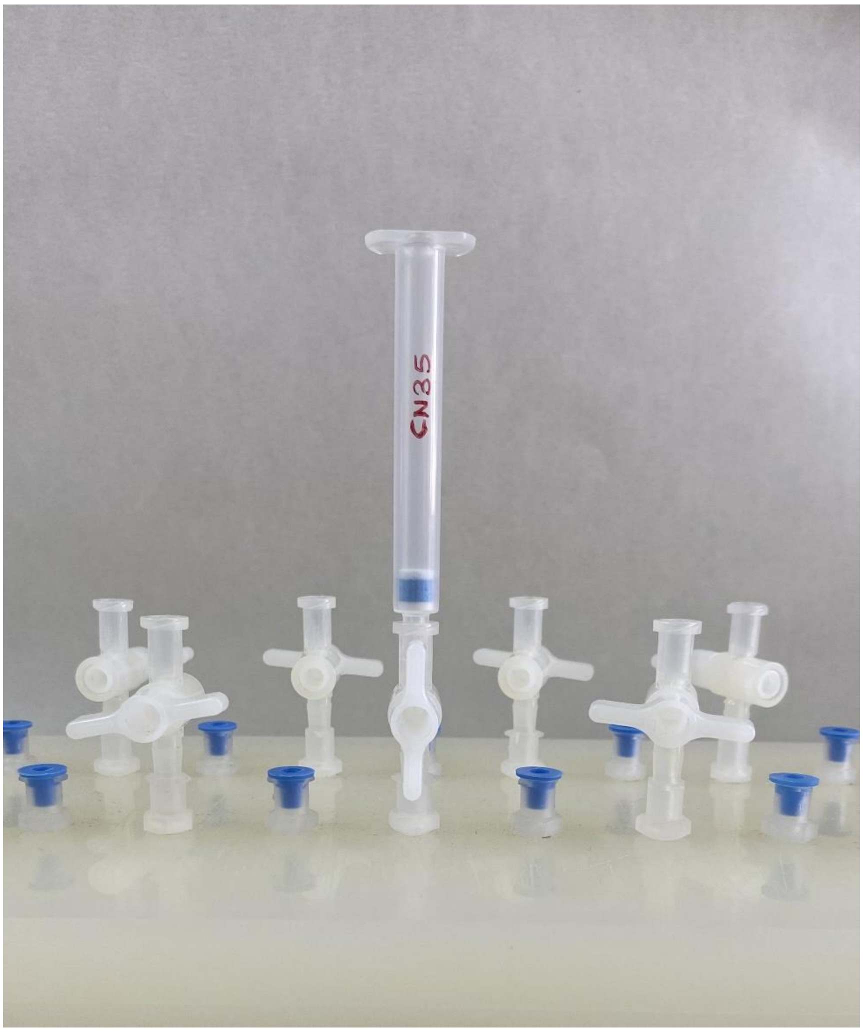

**Figure S1.** Image of the capture device incorporating SPE cartridges.

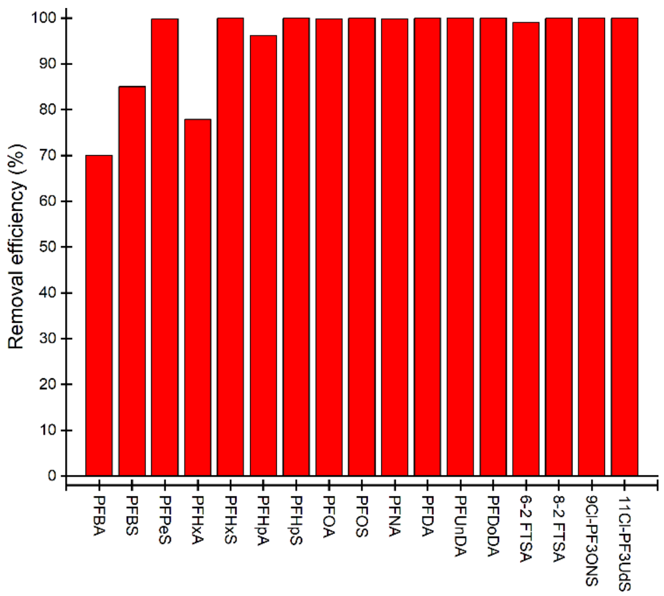


**Figure S2.** Capture efficiency (%) of MOF **1** from an aqueous solution containing a mixture of selected PFAS.

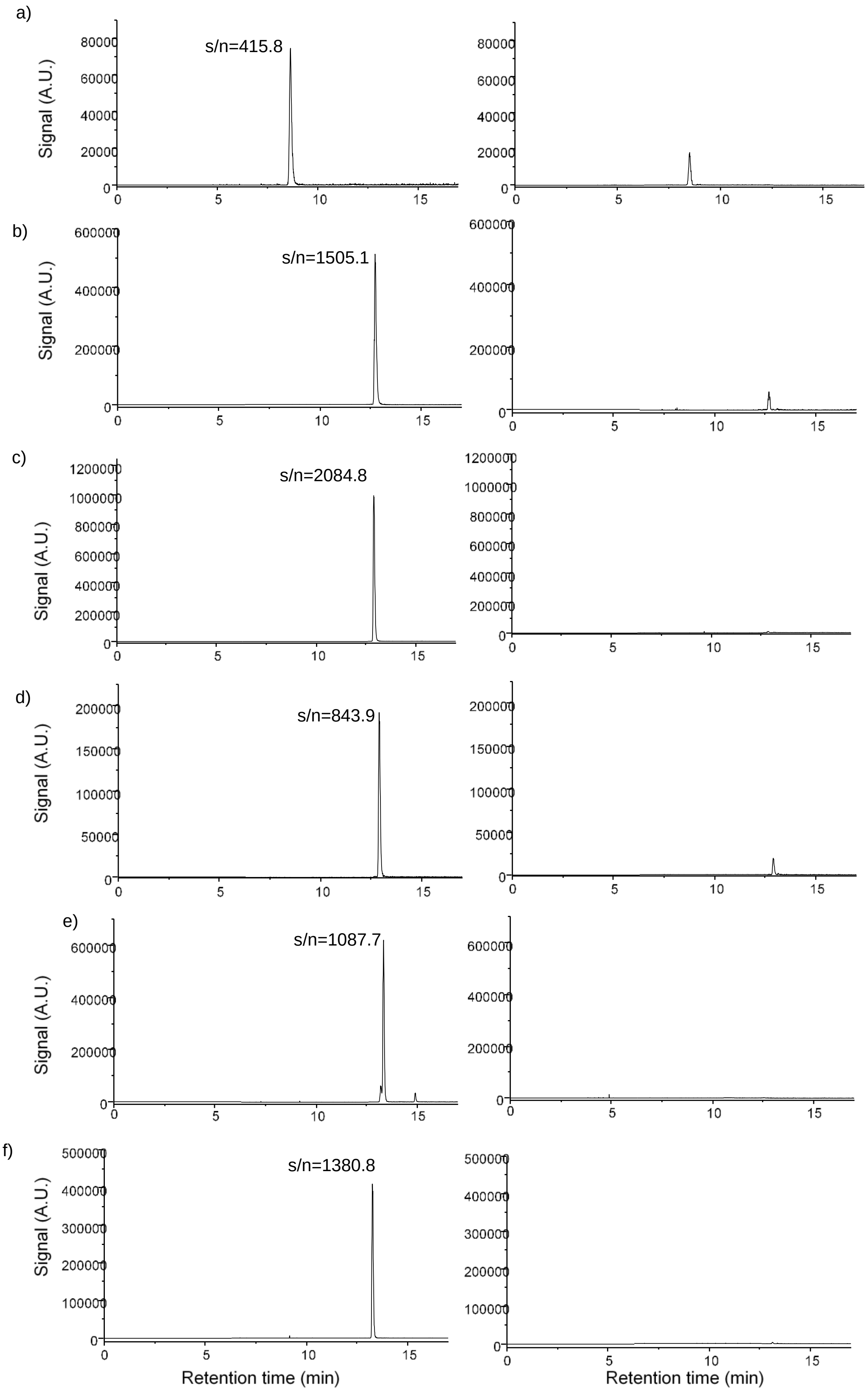
a)
s/n=415.8
b)
s/n=1505.1
c)
s/n=2084.8
d)
s/n=843.9
e)
s/n=1087.7
f)
s/n=1380.8
Signal (A.U.)
Retention time (min)
Retention time (min)

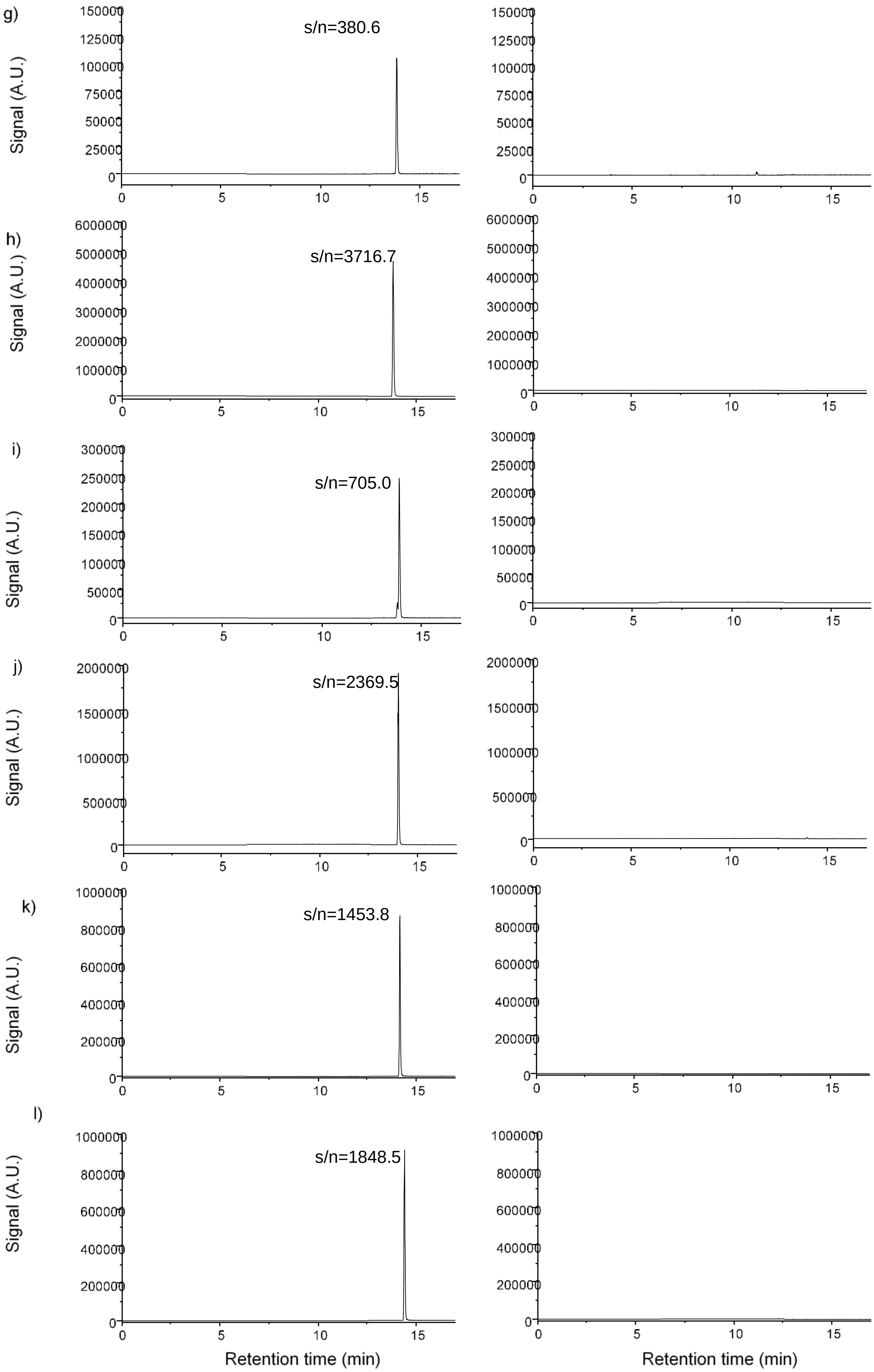
g)
s/n=380.6
h)
s/n=3716.7
i)
s/n=705.0
j)
s/n=2369.5
k)
s/n=1453.8
l)
s/n=1848.5
Signal (A.U.)
Retention time (min)
Retention time (min)

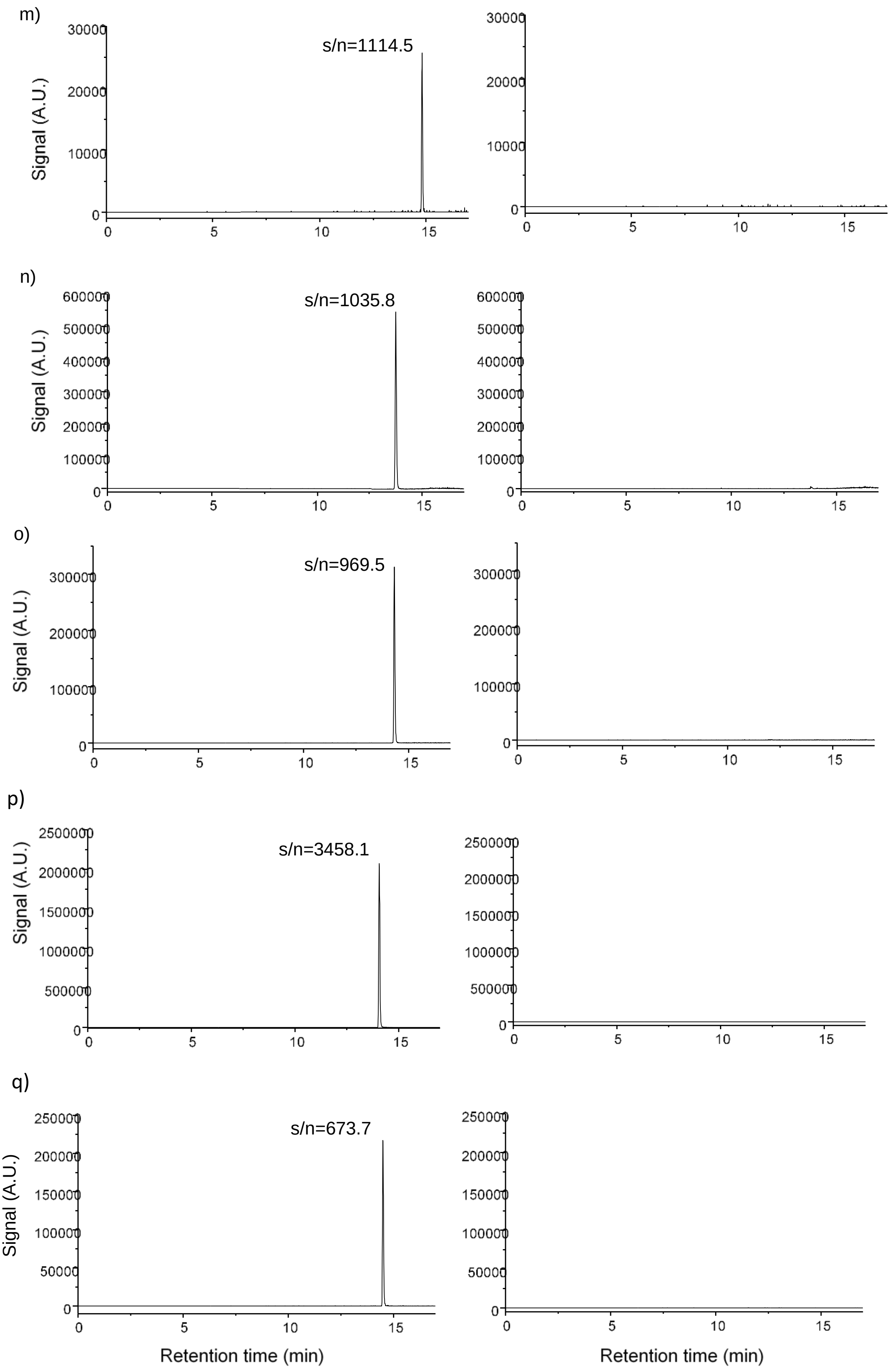


**Figure S3.** HPLC-MS spectra for each PFAS before (left) and after (right) the adsorption by MOF **1**. PFBA (a), PFBS (b), PFPeS (c), PFHxA (d), PFHxS (e), PFHpA (f), PFHpS (g), PFOA (h), PFOS (i), PFNA (j), PFDA (k), PFUnDA (l), PFDoDA (m) 6-2 FTSA (n), 8-2 FTSA (o), 9Cl-PF3ONS (p) and 11Cl-PF3OUdS (q).

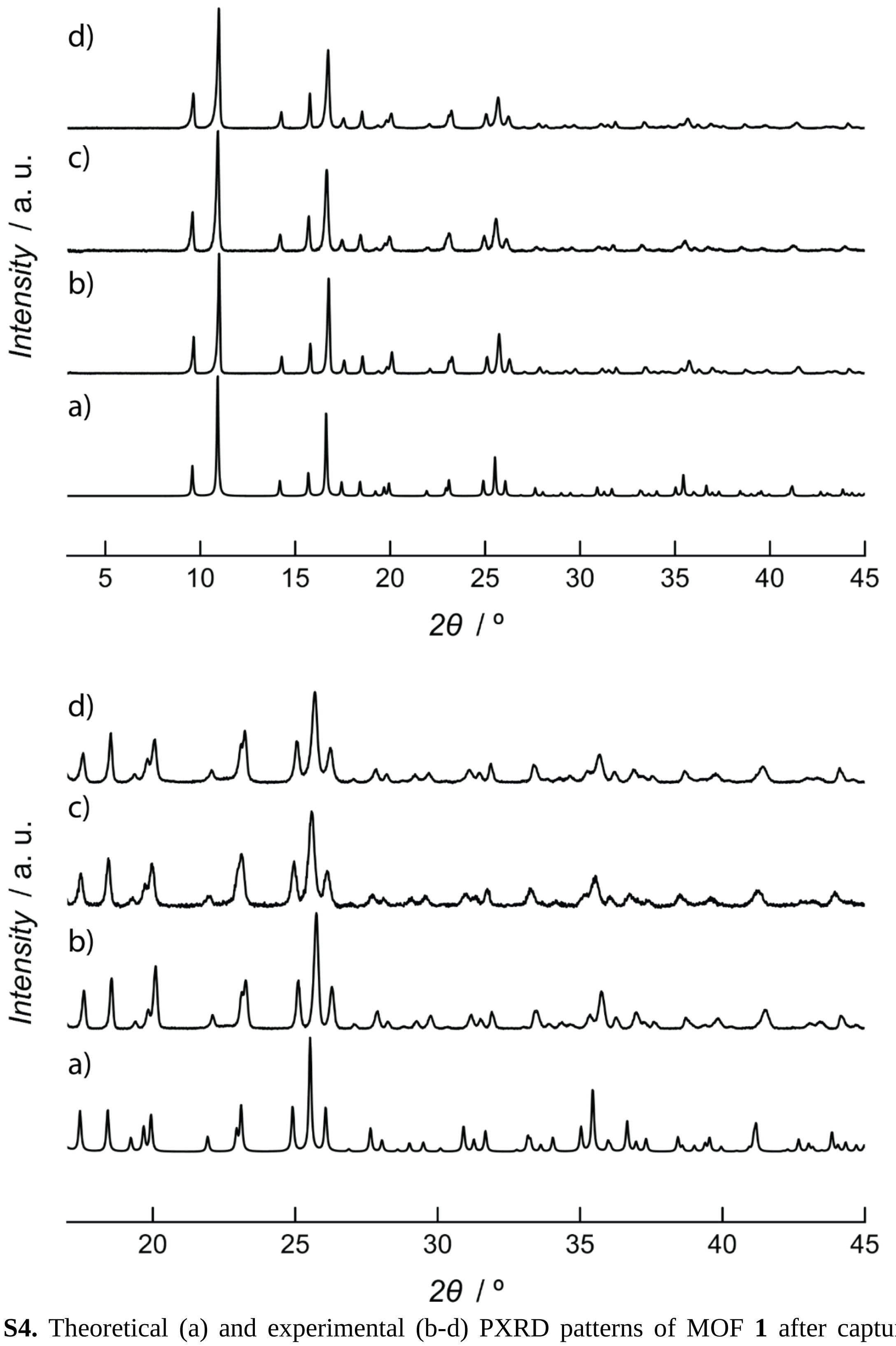


**Figure S4.** Theoretical (a) and experimental (b-d) PXRD patterns of MOF **1** after capture experiments at pH = 7 (b), 4 (c) and 10 (d) in the 2.0–45° (top) and 17–45° (bottom) ranges.

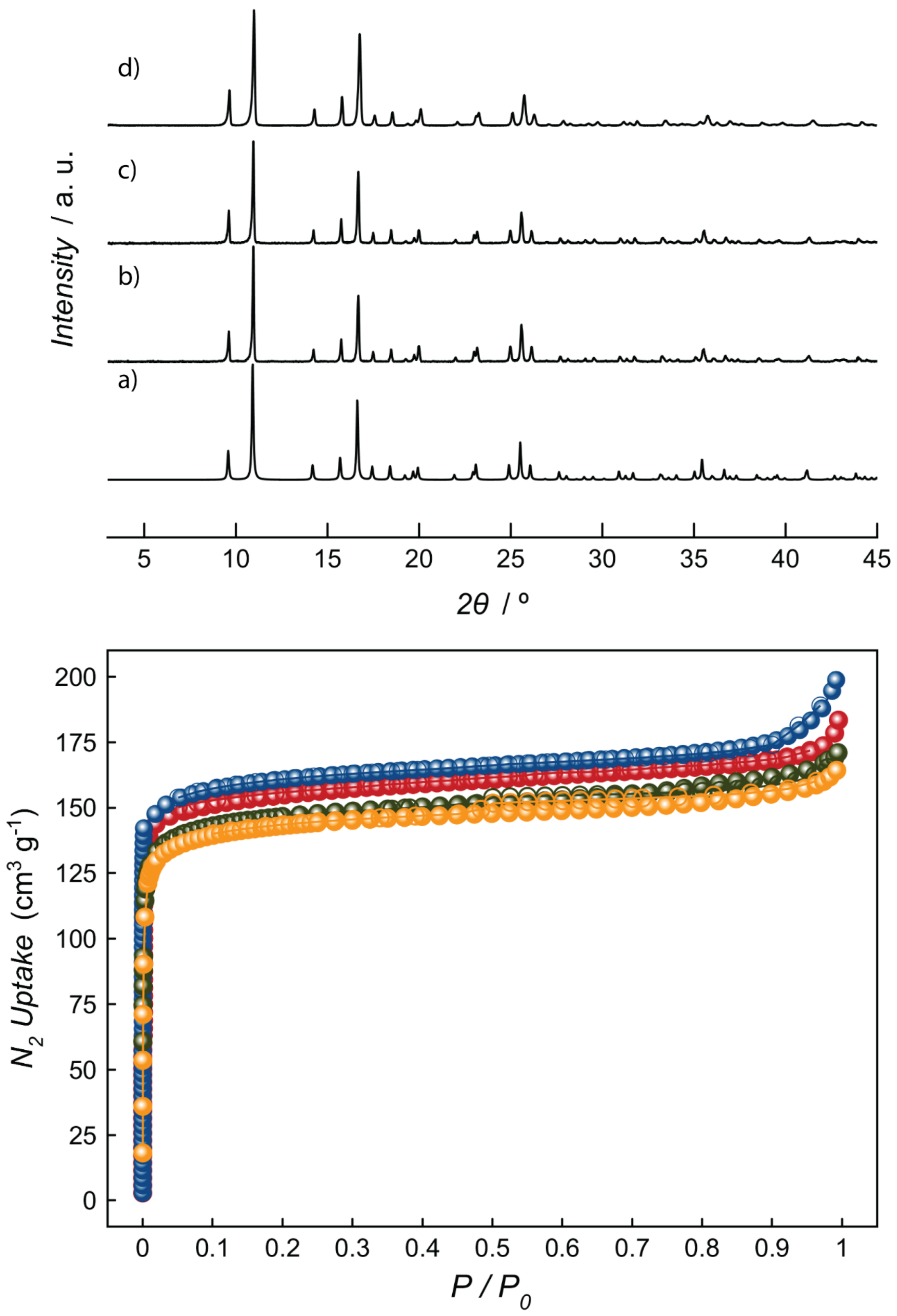


**Figure S5.** Top: Theoretical (a) and experimental (b-d) PXRD patterns of MOF **1** after being suspended in pH = 7 (b), pH = 4 (c) and pH = 10 (d) aqueous solutions for 14 days. Bottom: $N_2$ (77 K) adsorption isotherms for the activated MOF **1** before (red) after being suspended in pH = 7 (blue), pH = 4 (green) and pH = 10 (orange) aqueous solutions for 14 days. Filled and empty symbols indicate the adsorption and desorption isotherms, respectively. The samples were activated at 70 °C under reduced pressure for 16 h prior to carry out the sorption measurements.

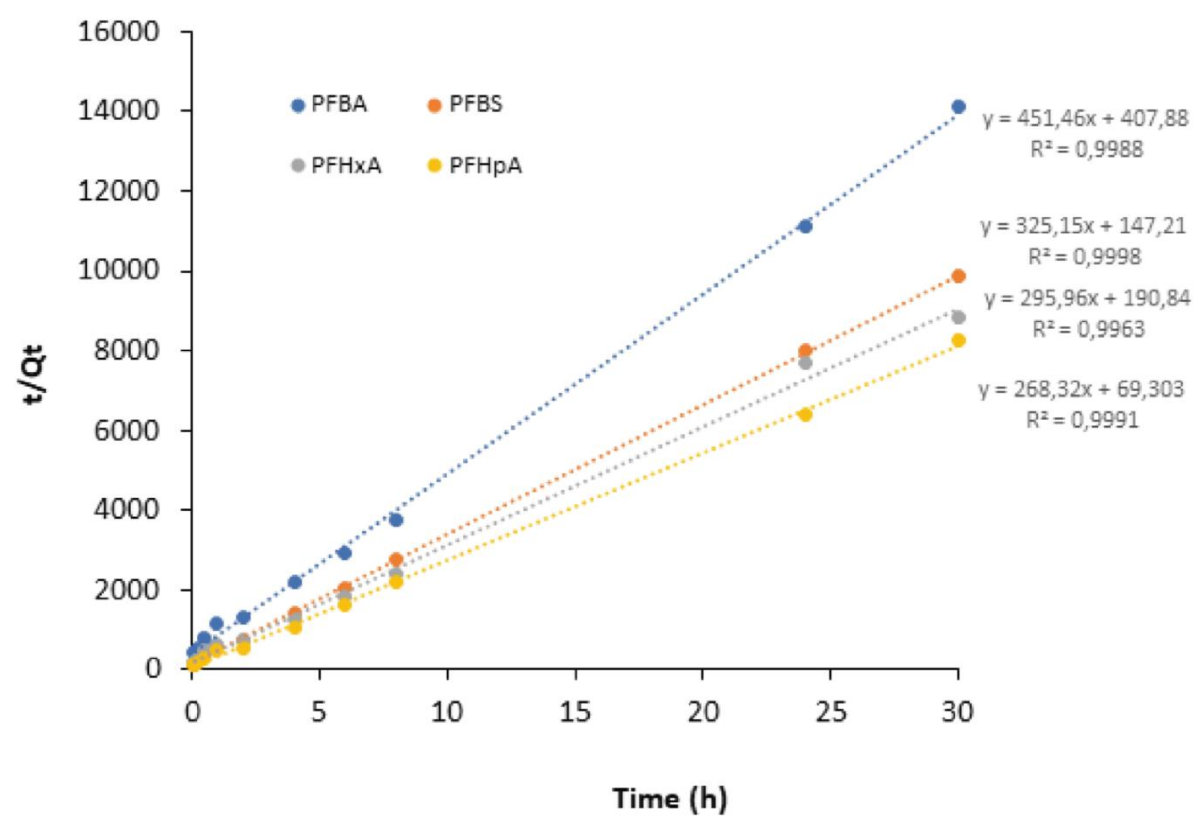


***Summary of kinetic parameters for pseudo-second order model***

| | Slope $1/Qe$ | Intercept $1/k_2(Qe)^2$ | Qe calc. (mg/g) | $k_2$ (g mg$^{-1}$ h$^{-1}$) | $R^2$ |
|---|---|---|---|---|---|
| **PFBA** | 451,46 | 407,88 | 0,0022 | 499,7 | 0,9988 |
| **PFBS** | 325,15 | 147,21 | 0,0031 | 718,2 | 0,9998 |
| **PFHxA** | 295,96 | 190,84 | 0,0034 | 459,0 | 0,9963 |
| **PFHpA** | 268,32 | 69,303 | 0,0037 | 1038,9 | 0,9991 |

***Summary of kinetic parameters for pseudo-first order model***

| | Slope $k_1$ (h$^{-1}$) | Intercept log Qe | Qe calc. (mg/g) | $R^2$ |
|---|---|---|---|---|
| **PFBA** | 0,2268 | -2,7093 | 0,00195 | 0,9915 |
| **PFBS** | 0,2022 | -2,8165 | 0,00153 | 0,8749 |
| **PFHxA** | 0,1945 | -2,6449 | 0,00227 | 0,9419 |
| **PFHpA** | | | | |

**Figure S6.** Kinetic models for compounds PFBA, PFBS, PFHxA, and PFHpA with a summary of kinetic parameters for pseudo-second and pseudo-first order model.

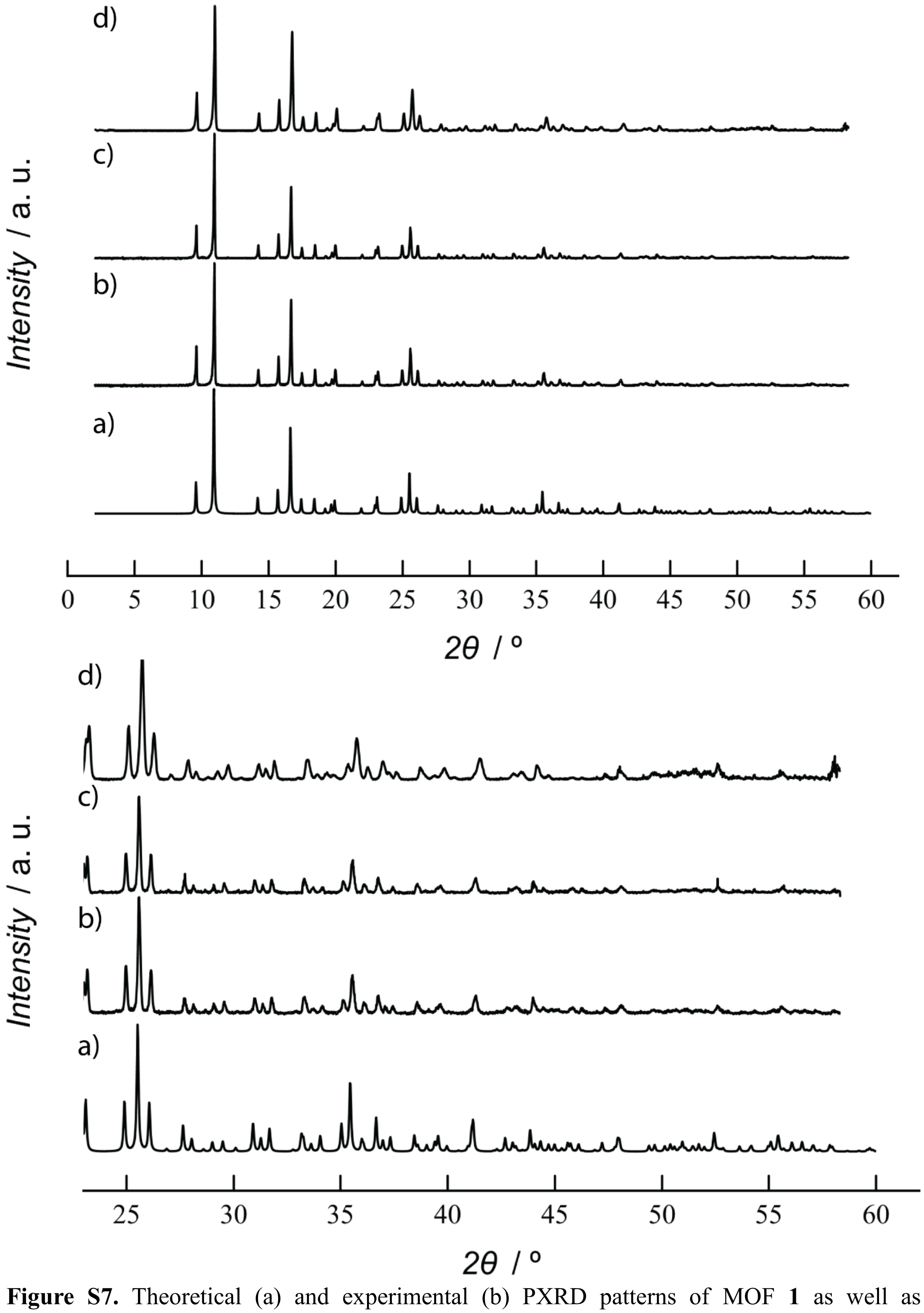


**Figure S7.** Theoretical (a) and experimental (b) PXRD patterns of MOF **1** as well as experimental PXRD patterns of MOF **1** after 15 consecutive reuse cycles (c) and after being suspended in water for 7 days (d), in the 2.0–60.0° (top) and 23.0–60.0° (bottom) ranges.

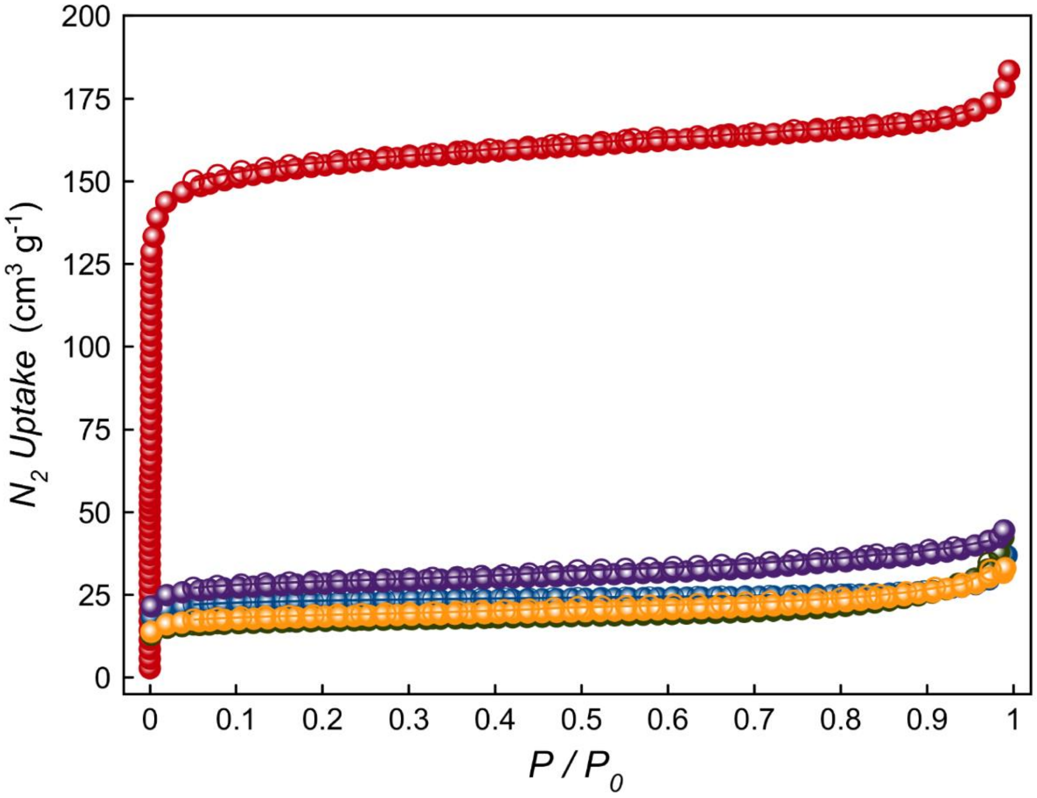


**Figure S8.** $N_2$ (77 K) adsorption isotherms for the activated compounds 1 (red) and the adsorbates **1+PFBS** (purple), **1+PFPeS** (blue), **1+PFHpS** (green), and **1+PFOS** (orange). Filled and empty symbols indicate the adsorption and desorption isotherms, respectively. The samples were activated at 70 °C under reduced pressure for 16 h prior to carry out the sorption measurements.

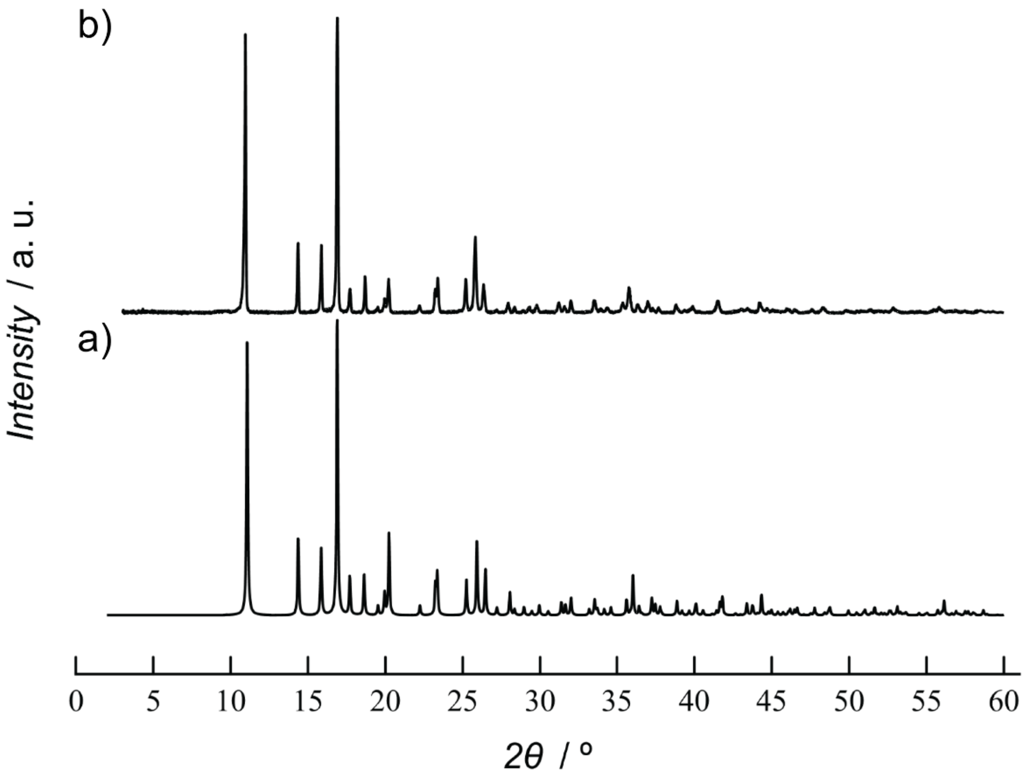


**Figure S9.** Theoretical (a) and experimental (b) PXRD patterns of **PFBA@1** in the 2.0–60.0° range.

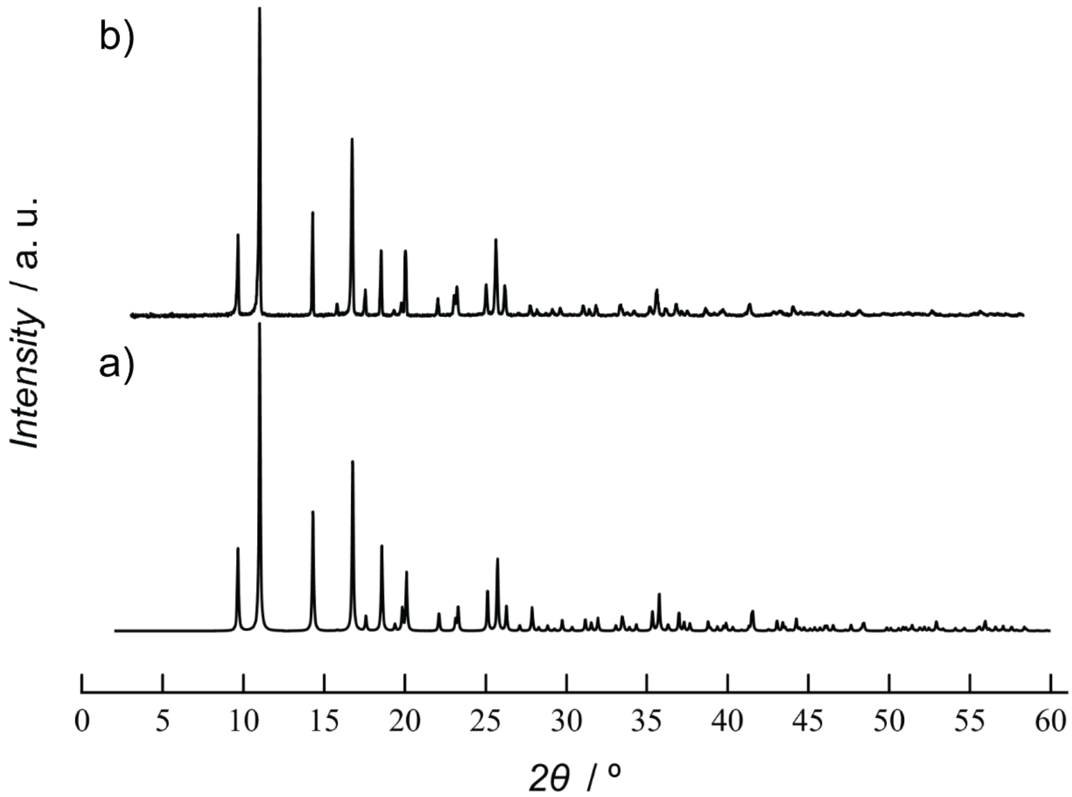


**Figure S10.** Theoretical (a) and experimental (b) PXRD patterns of **PFOS@1** in the 2.0–60.0° range.

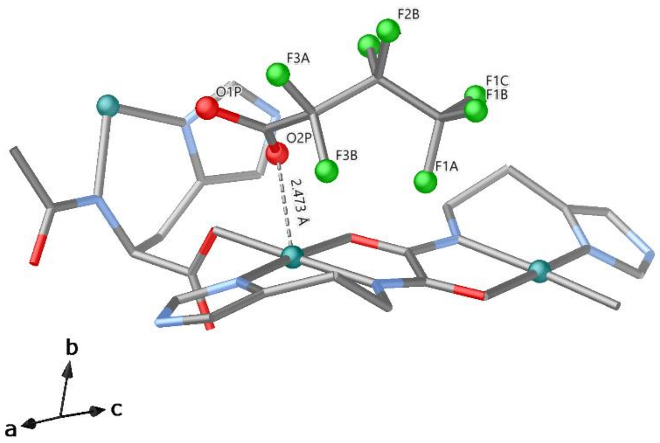


**Figure S11.** View of a fragment of the crystal structure of **PFBA@1**, highlighting the most stable conformation of PFBA guest molecules coordinated to the open copper metal site via their sulfonic groups (Cu···O interaction represented by a dashed line). Copper metal ions are depicted as cyan spheres, while the organic ligand is represented using a stick model. The PFBA guest molecules are shown with a ball-and-stick model. Color code: gray for carbon atoms, light blue for nitrogen, red for oxygen, and green for fluorine.

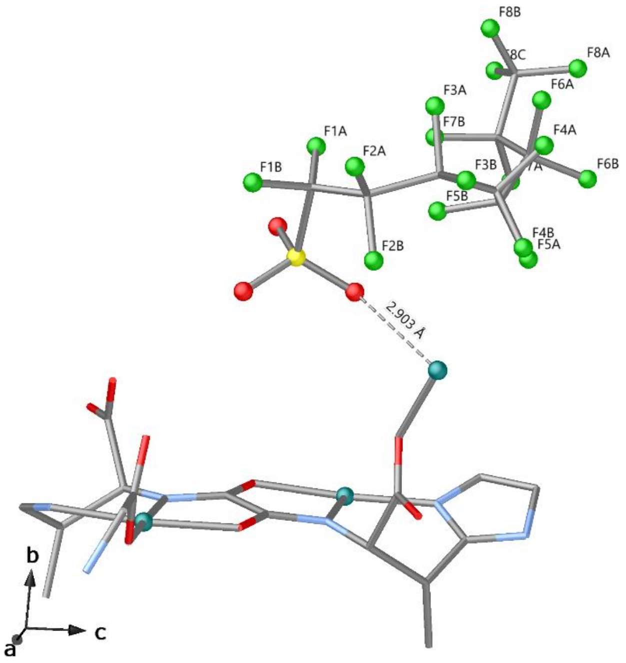


**Figure S12.** View of a fragment of the crystal structure of **PFOS@1**, highlighting the most stable conformation of PFOS guest molecules coordinated to the open copper metal site via their sulfonic groups (Cu···O interaction represented by a dashed line). Copper metal ions are depicted as cyan spheres, while the organic ligand is represented using a stick model. The PFOS guest molecules are shown with a ball-and-stick model. Color code: gray for carbon atoms, light blue for nitrogen, red for oxygen, green for fluorine, and yellow for sulfur.

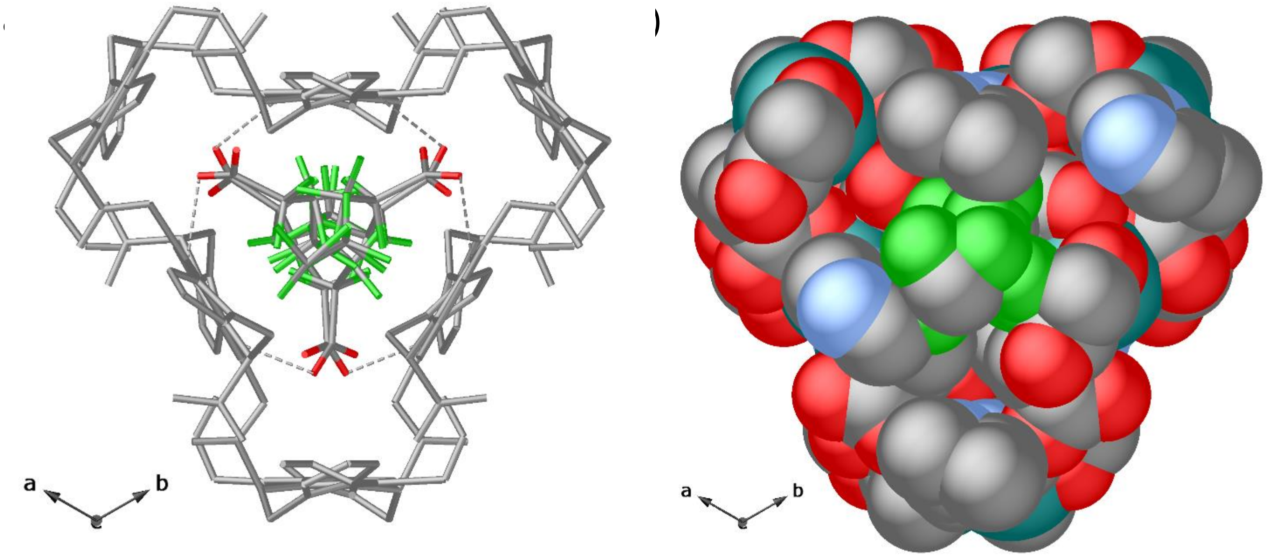


**Figure S13.** Perspective views in single channels generated in crystal structures of **PFBA@1** with stick (a) and space filling models (b). Color code: gray for carbon atoms [in the stick model depicted in a) gray is for all atoms of the MOF networks]; light blue for nitrogen, red for oxygen, and green for fluorine.

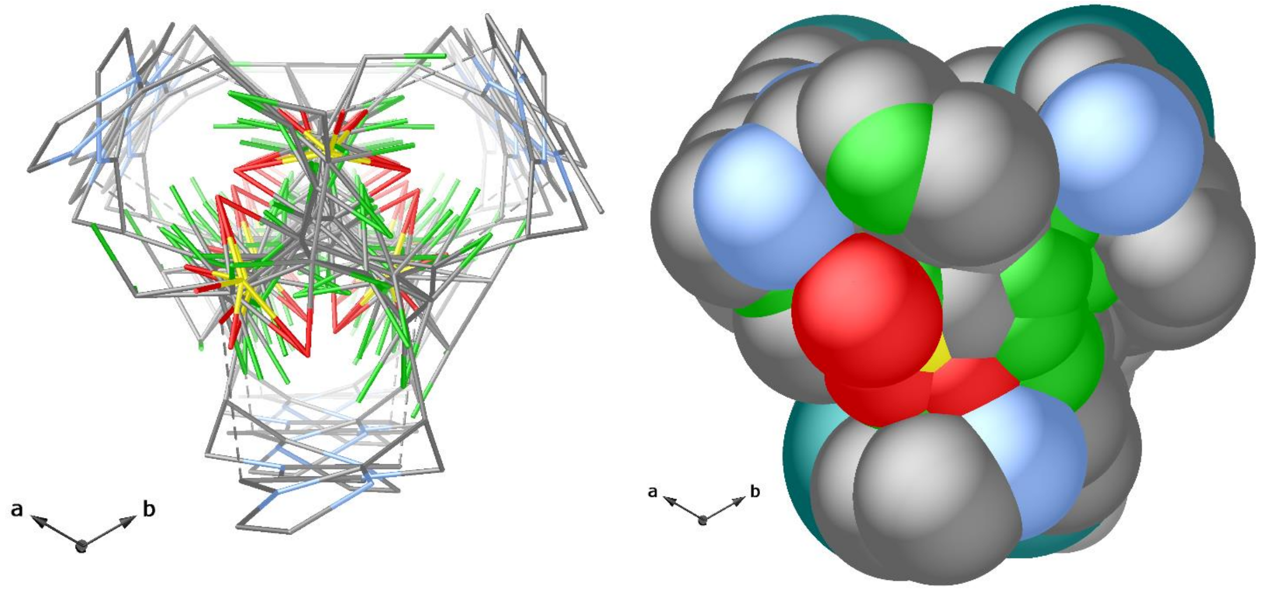


**Figure S14.** Perspective views in single channels generated in crystal structures of **PFOS@1** with stick (a) and space filling models (b). Color code: gray for carbon atoms [in the stick model depicted in a) gray is for all carbon and oxygen atoms of the MOF networks]; light blue for nitrogen, red for oxygen, yellow for sulfur, and green for fluorine.

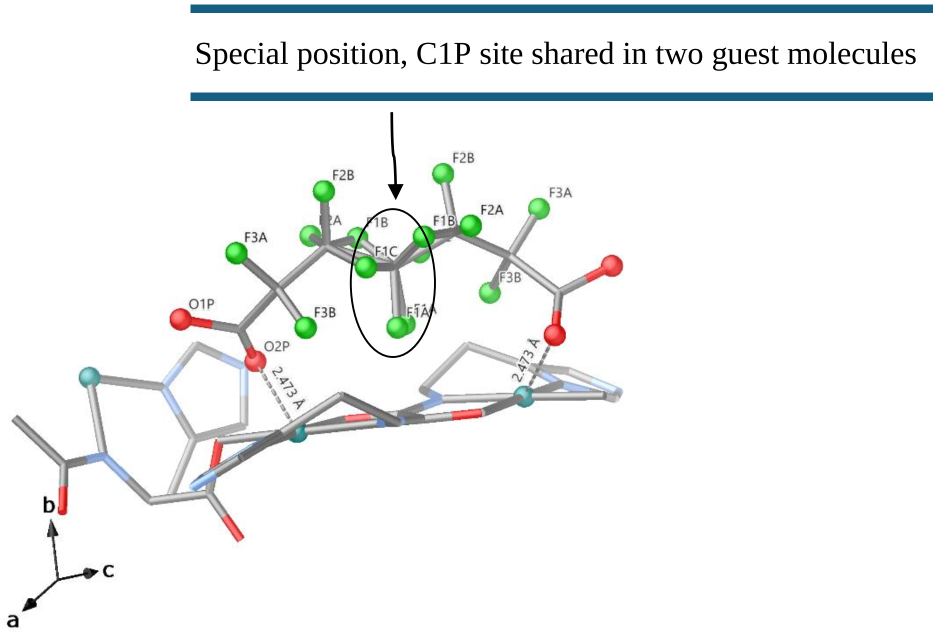


**Figure S15.** View of statistical disorder of PFBA guest molecules in **PFBA@1**. Copper metal ions are depicted as cyan spheres, while the organic ligand is represented using a stick model. The PFBA guest molecules are shown with a ball-and-stick model. Color code: gray for carbon atoms, light blue for nitrogen, red for oxygen, green for fluorine, and yellow for sulfur.

**References**

[1] M. Mon, R. Bruno, E. Tiburcio, A. Grau-Atienza, A. Sepúlveda-Escribano, E. V. Ramos-Fernandez, A. Fuoco, E. Esposito, M. Monteleone, J. C. Jansen, et al., *Chem. Mater.* **2019**, *31*, 5856–5866.

[2] SAINT, version 6.45, Bruker Analytical X-ray Systems, Madison, WI, 2003.

[3] Sheldrick G.M. SADABS Program for Absorption Correction, version 2.10, Analytical X-ray Systems, Madison, WI, 2003.

[4] (a) Sheldrick, G. M. Crystal structure refinement with SHELXL. *Acta Cryst. C* **71**, 3–8 (2015). (b) Sheldrick, G. M. A short history of SHELX. *Acta Cryst. A* **64**, 112–122 (2008). (c) SHELXTL-2013/4, Bruker Analytical X-ray Instruments, Madison, WI, 2013.

[5] (a) Spek, A. L. *PLATON* SQUEEZE: a tool for the calculation of the disordered solvent contribution to the calculated structure factors. *Acta Crystallogr. Sect. C-Struct. Chem.* **71**, 9–18 (2015). (b) Spek, A. L. Structure validation in chemical crystallography. *Acta Crystallogr. Sect. D, Biol. Crystallogr.* **65**, 148–155 (2009).

[6] Farrugia, L. J. *WinGX* suite for small-molecule single-crystal crystallography. *J. Appl. Crystallogr.* **32**, 837–838 (1999).

[7] Palmer, D. CRYSTAL MAKER, Cambridge University Technical Services, C. No Title, 1996.
